# Supplementary material for: ISA API: An open platform for interoperable life science experimental metadata
Source: Gigascience. 2021 Sep 16;10(9):giab060. doi: 10.1093/gigascience/giab060 (PMC8444265; doi:10.1093/gigascience/giab060)

GigaScience

ISA API: An open platform for interoperable life science experimental metadata

--Manuscript Draft--

**Manuscript Number:**	GIGA-D-20-00333R1
**Full Title:**	ISA API: An open platform for interoperable life science experimental metadata
**Article Type:**	Technical Note
**Funding Information:**	Horizon 2020 (EC654241) — Not applicable<br>Biotechnology and Biological Sciences Research Council (BB/L021390/1 [BB/L024055/1, BB/L024071/1, BB/L024101/1]) — Not applicable<br>Biotechnology and Biological Sciences Research Council (BB/J020265/1) — Not applicable<br>Biotechnology and Biological Sciences Research Council (BB/M027635/1) — Not applicable<br>Natural Environment Research Council (NE/L002493/1) — Not applicable

Funder	
Horizon 2020 (EC654241)	Not applicable
Biotechnology and Biological Sciences Research Council (BB/L021390/1 [BB/L024055/1, BB/L024071/1, BB/L024101/1])	Not applicable
Biotechnology and Biological Sciences Research Council (BB/J020265/1)	Not applicable
Biotechnology and Biological Sciences Research Council (BB/M027635/1)	Not applicable
Natural Environment Research Council (NE/L002493/1)	Not applicable

Abstract:

Background

The Investigation/Study/Assay (ISA) Metadata Framework is an established and widely used set of open-source community specifications and software tools for enabling discovery, exchange and publication of metadata from experiments in the life sciences. The original ISA software suite provided a set of user-facing Java tools for creating and manipulating the information structured in ISA-Tab – a now widely used tabular format. To make the ISA framework more accessible to machines and enable programmatic manipulation of experiment metadata, a JSON serialization ISA-JSON was developed.

Results

In this work, we present the ISA API, a Python library for the creation, editing, parsing, and validating of ISA-Tab and ISA-JSON formats by using a common data model engineered as Python class objects. We describe the ISA API feature set, early adopters and its growing user community.

Conclusions

The ISA API provides users with rich programmatic metadata handling functionality to support automation, a common interface and an interoperable medium between the two ISA formats, as well as with other life science data formats required for depositing data in public databases.

**Corresponding Author:**	Philippe Rocca-Serra<br>University of Oxford<br>Oxford, Oxfordshire UNITED KINGDOM
**Corresponding Author Secondary Information:**	
**Corresponding Author's Institution:**	University of Oxford
**Corresponding Author's Secondary Institution:**	
**First Author:**	David Johnson
**First Author Secondary Information:**	
**Order of Authors:**	David Johnson
	Keeva Cochrane

Powered by Editorial Manager® and ProduXion Manager® from Aries Systems Corporation

	Robert P Davey
	Anthony Etuk
	Alejandra Gonzalez-Beltran
	Kenneth Haug
	Massimiliano Izzo
	Martin Larralde
	Thomas N Lawson
	Alice Minotto
	Pablo Moreno
	Venkata Chandrasekhar Nainala
	Claire O'Donovan
	Luca Pireddu
	Pierrick Roger
	Felix Shaw
	Christoph Steinbeck
	Ralf JM Weber
	Susanna-Assunta Sansone
	Philippe Rocca-Serra
**Order of Authors Secondary Information:**	
**Response to Reviewers:**	Editor comment: Your manuscript "ISA API: An open platform for interoperable life science experimental metadata" (GIGA-D-20-00333) has been assessed by our reviewers. Although it is of interest, we are unable to consider it for publication without some additional work. The reviewers have raised a number of points which we believe would improve the manuscript and would allow a revised version to be published in GigaScience.<br><br>Author response: First, we thank the reviewers for their time and expertise to appraise our work and we are grateful for the opportunity given to improve our manuscript, software and to clarify any misunderstandings. We enclose henceforth our responses to each of the comments, as well as indications of the changes, where we agree a change is warranted, to the initial manuscript to address specific comments.<br><br>Editor comment: In addition, please register any new software application in the bio.tools and SciCrunch.org databases to receive RRID (Research Resource Identification Initiative ID) and biotoolsID identifiers, and include these in your manuscript. This will facilitate tracking, reproducibility and re-use of your tool.<br><br>Author response: We have added the biotoolsID and SciCrunch RRID to the “Availability of source code and requirements” section found on page 20.<br><br>Editor comment: Please include a point-by-point within the 'Response to Reviewers' box in the submission system. Please ensure you describe additional experiments that were carried out and include a detailed rebuttal of any criticisms or requested revisions that you disagreed with. Please also ensure that your revised manuscript conforms to the journal style, which can be found in the Instructions for Authors on the journal homepage. If the data and code has been modified in the revision process please be sure to update the public versions of this too.<br><br>Author response: In our ‘Response to Reviewers’ we describe our rebuttals of any points we disagreed with and also point to the pages where we made changes in the draft manuscript for the points we do agree with. The revised manuscript remains styled as per the Instructions to Authors. No data and code has been modified in the

revision process.

Responses to Reviewer 1
Reviewer 1 remarks: In the paper "ISA API: An open platform for interoperable life science experimental metadata" Johnson et al. present a extensive Python API for reading, writing and handling of metadata in the ISA format. The authors describe the increasing use of the ISA formats and thus indicate the need for better tools to handle such data. The article is well written and good to understand.

The ISA tools package contains extensive functionality and a solid documentation. Furthermore, it can be installed with PyPi and Bioconda, which I think should be standard nowadays. The authors furthermore provide a docker image, which is nice. All in all, I think the ISA tools package is a genuinely useful piece of software that is well written, which is why I recommend this manuscript for publication in GigaScience.

Author response: We appreciate Reviewer 1's comments and are heartened that they felt the article is well written and recommended for publication in GigaScience. Below we address their comments point-by-point:

Reviewer 1 comment: I would like to know whether support for the upload to additional databases will be added in the future - this could be noted in the text.

Author response: As a free and open source software library, an objective of ISA API is to develop any new features the ISA user community proposes. Beyond the current export functionality, we fully expect to continue supporting data deposition to EMBL-EBI data repositories and also are working in close collaboration with FAIRdom/SEEK.

Author changes: We prefer not to speculate on future work too specifically in the manuscript, but we have added a line to state "Support for submission to additional databases will be developed in future" on page 8.

Reviewer 1 comment: The article contains many figures with only little content. I would strongly advise to merge some of these figures into a smaller subset of figures to improve the readability.

Author response: We have reviewed the manuscript's figures. Figures 1-5 contain text and we believe those figures would not be very readable if reduced. We think this can be addressed further if necessary in the typesetting phase of manuscript production, as the draft manuscript is presented without any typeset formatting as per the GigaScience manuscript preparation guidelines in https://academic.oup.com/gigascience/pages/instructions_to_authors#Preparing%20Main%20Manuscript%20Text.

Author changes: We have merged figures 6 and 7 (download stats) into a single figure as two side-by-side panels found on page 15.

Reviewer 1 comment: The authors spend a considerable amount of text on download statistics - something that in my opinion is not really that relevant for the software package. I would recommend to considerably shorten this section.

Author changes: We have now considerably shortened this section on pages 15 and 16.

Reviewer 1 comment: On a similar note, the methods section basically just describes how these download statistics were handled. Considering this article describes a software package, it might be more useful to the reader (and reviewer) to elaborate a bit on how the software is written, maintained, structured, tested - and related things.

Author changes: We have added a discussion about our development approaches in the "Methods" section of the manuscript on pages 17-19 and also about ISA API's documentation on page 13.

Responses to Reviewer 2
Reviewer 2 remarks: The authors describe the Python library "isatools" for accessing

Powered by Editorial Manager® and ProduXion Manager® from Aries Systems Corporation

ISA (investigation study assay) files in ISA-tab and ISA-json format. The authors start by sdescribing their previous work around the ISA data model and file formats in detail. They then describe their implementation and the features of their API. They highlight the extensibility and efficiency of their object oriented model. They describe in detail how meta data can be curated in ontologies and that currently extensions are underway for the assisted creation of study meta data. They then refer to early adopters and a stable and growing community. They conclude with the statement that their library is "a major step forward in making the ISA framework open and interoperable".

Overall, we have found the ISA data model and ISA-tab data format to be very useful in our own work. However, there are some issues with the software including apparent bugs as described below. In 2018, my colleagues and me considered using ISA-API in our project for ISA-Tab parsing but the problems and the lack of automated tests made us roll our own (also see below).

Overall, the authors make a clear point, the paper is well-written. However, the software appears to be unfinished and some work is required to make it suitable for publication.

Author response: We appreciate Reviewer 2's comments on the manuscript and for sharing their opinions about the software based on their personal experiences, and are happy to hear that they state that the paper is well-written. We disagree that the software is unfinished and rebut the reviewer's specific points on that issue in our responses below.

Below we address their specific comments point-by-point:

Reviewer 2 comment: The ISA-creator and Bio-GraphIIn are cited as "helped grow the ISA community of users". The authors should offer evidence for this as

(a) by our own experience ISA-creator is very hard to use and this is also reflected by the expressed opinion on ISA-creator by anyone I have met so far who has used it and

(b) it is not possible to validate how Bio-GraphII has helped grow the community as the website linked to in the cited article is not available anymore and no source code is available, e.g., on Github. The Google groups forum has less than 10 threads per year, with 2 in 2020 so far and one in 2019. The authors should balance these counts with their "PyPi" download counts statistics.

Author response: ISAcreator has been the de facto reference implementation of the ISA-Tab specification. From our own experience, we know that researchers have been consistently using ISAcreator over many years to prepare their data submissions for various databases. This includes Metabolights (EMBL-EBI), GenomeSpace (Broad Institute), Toxbank (EU), and Genelab (NASA) to name just a few.

We agree that Bio-GraphIIn should in fact not be mentioned as it has been long retired. This should instead have referenced the BioInvestigation Index (BII) database software, which has been used by various organisations worldwide including the UK NERC Environmental Bioinformatics Centre, the US Personalized NSAID Therapeutics Consortium (PENTACON), and the Harvard Stem Cell Discovery Engine. There is evidence of BII still in use - the aforementioned organisations continue to maintain running instances.

The known ISA community is listed in the ISA Commons, which is referenced in the manuscript.

We disagree that Google groups activity can be indicative of ISA community activity. The ISA Google group has historically had low activity owing to it being a secondary point of contact to the direct email address to the ISA team.

Author changes: We have replaced reference to Bio-GraphIIn with BioInvestigation Index on page 5.

Powered by Editorial Manager® and ProduXion Manager® from Aries Systems Corporation

Reviewer 2 comment: The authors should cite other published APIs for ISA file formats, e.g., AltamISA.

- Kuhring, Mathias, et al. "AltamISA: a Python API for ISA-Tab files." Journal of Open Source Software 4.40 (2019): 1610.

Author response: We agree that references to other published ISA-related software should be included and appreciate that the reviewer suggests that we cite his own work.

Author changes: We additionally believe that we should make reference to precursor Perl and Python projects that we are also aware of. We have added a new “Related work” subsection at the end of the Background section beginning on page 6 that references Bio-Parser-ISATab, biopy-isatab, AltamISA and isa4j.

Reviewer 2 comment: The authors should show proof for "efficiency" of their object-oriented model, e.g., by comparing import efficiency with that of altamISA. I'm raising this point as some users raised questions on efficiency when loading/writing data files in the ISA-API Github Issues.

Author response: The use of the word “efficient” in the manuscript is about the representation of ISA content in the data model and not about the processing performance (ie. when doing i/o). We do not intend to present in this paper any performance evaluation/comparison of ISA API with other software that is not comparable in terms of features. As far as we understand, AltamISA and isa4j focus only on ISA-Tab. ISA API has a much broader scope to interoperate between ISA formats and other formats.

Author changes: We have updated the single use of the word “efficient” to “coherent” in the relevant subsection heading on page 9 to avoid confusion to the reader. Efficiency is not discussed in the manuscript.

Reviewer 2 comment: The authors write that development is in progress but it appears from the Github code frequency graph that development has mostly stalled since 2018.

Author response: We disagree that development has stalled since 2018 and disagree that the GitHub code frequency graph is a good measure of development.

The GitHub code frequency graph is unrepresentative of activity as it is weighted on the numbers of lines of code (LOC) added/removed. LOC may indicate the degree of changes to source code but we believe that even small changes indicate that a software is being actively maintained or updated. A better view of activity may be the graph of contributions to the master branch (https://github.com/ISA-tools/isa-api/graphs/contributors) that counts commits rather than LOC and clearly shows activity since 2018.

We also believe development work goes beyond activity shown by GitHub commits, such as testing, feature requests, and collaborating with the user community who are using/developing with ISA API as development, activity of which is not always reflected on GitHub.

We can confirm to the reviewer that development continues and has not stalled since 2018.

Reviewer 2 comment: The authors should explain in more detail how stable their API is and what the limitations and assumptions are. In my opinion, one important point in data import and export is looking how data looks after a "round-trip", e.g., import ISA-Tab, followed by export ISA-Tab.

I have done this on the official ISA data sets (https://github.com/ISA-tools/ISAdatasets, commit f20be4f83dc5f6f7ec419bfd634efba3177e4ae4). Here are the (to me unexpected results for official example data):

(a) On BII-I-1, whole columns disappear such as the first "Material Type" column,

	(b) All other datasets fail to parse and parsing crashes with Python exceptions.<br><br>I think the authors should work on these points. It cannot be judged whether the software can be published this point. The software appears unfinished and some more work has to go into it to allow for publication.<br><br>Author response: We disagree that the software appears unfinished.<br><br>While we appreciate that the reviewer has carried out their own round-tripping tests, we cannot respond to an informally conducted test with very few details revealed to us. We can point out to the reviewer that the ISA datasets commit they reference in their review is not on the test data branch of which ISA API uses in the project's automated test suite. Round-tripping only works as a test if you assume the input is valid to begin with. Additionally, round-tripping is a narrow case that does not represent the lion's share of use cases for ISA API, which is to interoperate ISA formats with other formats.<br><br>We encourage the reviewer to actively engage with the open source ISA API project to understand how ISA API's test infrastructure works, and so that they can report bugs, or contribute fixes if they have tried to fix any bugs. The project has been open source since its inception in 2015 and has always universally welcomed contributions/discussions with existing or potential users.<br><br>The point is therefore more an issue of documentation than implementation. Pretty much all projects can be faulted on the documentation. This is also to address some of these issues that the ISA API documentation has been migrated from readthedocs to the more modern jupyterbook infrastructure.<br><br>We strongly believe that based on our user community's adoption of the ISA API (the major adopters of which are described in the paper), and the sustained and growing number of downloads via PyPI, that the software is stable and being used in production by third parties.<br><br>Reviewer 2 comment: The authors should provide more automated tests for their software. In 2018 when we tried out the package we found some inconsistencies and problems but found it hard to fix bugs in the large body of software because of the lack of comprehensive automated tests.<br><br>Author response: We disagree that there is a lack of comprehensive automated tests and are sorry to hear that they found it hard to fix bugs in 2018.<br><br>Today, ISA API today has 631 automated unit tests on the master branch of the GitHub project. When bugs are reported, we usually add a test relating to the bug in our automated test suite to trace and fix bugs in a TDD process.<br><br>Where there are any issues found, we warmly welcome everybody to log any issues and make fix/feature requests, and to contribute/collaborate with fixes/features, via our open source GitHub project.<br><br>Author changes: We have added a discussion about our development approaches in the "Methods" section of the manuscript (also suggested by Reviewer 1) on pages 17-19.
**Additional Information:**	
**Question**	**Response**
Are you submitting this manuscript to a special series or article collection?	No
**Experimental design and statistics**<br><br>Full details of the experimental design and statistical methods used should be given	Yes

in the Methods section, as detailed in our Minimum Standards Reporting Checklist. Information essential to interpreting the data presented should be made available in the figure legends.<br><br>Have you included all the information requested in your manuscript?	
**Resources**<br><br>A description of all resources used, including antibodies, cell lines, animals and software tools, with enough information to allow them to be uniquely identified, should be included in the Methods section. Authors are strongly encouraged to cite Research Resource Identifiers (RRIDs) for antibodies, model organisms and tools, where possible.<br><br>Have you included the information requested as detailed in our Minimum Standards Reporting Checklist?	Yes
**Availability of data and materials**<br><br>All datasets and code on which the conclusions of the paper rely must be either included in your submission or deposited in publicly available repositories (where available and ethically appropriate), referencing such data using a unique identifier in the references and in the "Availability of Data and Materials" section of your manuscript.<br><br>Have you have met the above requirement as detailed in our Minimum Standards Reporting Checklist?	Yes

Powered by Editorial Manager® and ProduXion Manager® from Aries Systems Corporation

Manuscript

Click here to access/download;Manuscript;ISA API paper - GigaScience technical note-revisions.docx

ISA API: An open platform for interoperable life science experimental metadata

David Johnson[1,2,†], Keeva Cochrane[3], Robert P. Davey[4], Anthony Etuk[4], Alejandra Gonzalez-Beltran[1,5], Kenneth Haug[3,6], Massimiliano Izzo[1], Martin Larralde[7], Thomas N. Lawson[8], Alice Minotto[4], Pablo Moreno[3], Venkata Chandrasekhar Nainala[3], Claire O'Donovan[3], Luca Pireddu[9], Pierrick Roger[10], Felix Shaw[4], Christoph Steinbeck[11], Ralf J. M. Weber[8,12], Susanna-Assunta Sansone[1*,†], Philippe Rocca-Serra[1*,†]

[1]Oxford e-Research Centre, Department of Engineering Science, University of Oxford, 7 Keble Road, OX1 3QG, Oxford, United Kingdom, [2]Department of Informatics and Media, Uppsala University, Box 513, 751 20 Uppsala, Sweden, [3]European Molecular Biology Laboratory, European Bioinformatics Institute (EMBL-EBI), Wellcome Genome Campus, Hinxton, Cambridge CB10 1SD, United Kingdom, [4]Earlham Institute, Norwich Research Park, Norwich NR4 7UZ, United Kingdom, [5]Science and Technology Facilities Council, Scientific Computing Department, Rutherford Appleton Laboratory, Harwell Campus, Didcot, OX11 0QX, United Kingdom, [6]Genome Research Limited, Wellcome Sanger Institute, Wellcome Trust Genome Campus, Hinxton, Saffron Walden CB10 1RQ, United Kingdom, [7]Structural and Computational Biology Unit, European Molecular Biology Laboratory (EMBL), Meyerhofstraße 1, 69117 Heidelberg, Germany, [8]School of Biosciences, University of Birmingham, Edgbaston, Birmingham, B15 2TT, United Kingdom, [9]Distributed Computing Group, CRS4: Center for Advanced Studies, Research & Development in Sardinia, Pula, Italy, [10]CEA, LIST, Laboratory for Data Analysis and Systems' Intelligence, MetaboHUB, Gif-Sur-Yvette F-91191, France, [11]Cheminformatics and Computational Metabolomics, Institute for Analytical Chemistry, Lessingstr. 8, 07743 Jena, Germany, [12]Phenome Centre Birmingham, University of Birmingham, Edgbaston, Birmingham, B15 2TT, United Kingdom

*Corresponding authors: susanna-assunta.sansone@oerc.ox.ac.uk, philippe.rocca-serra@oerc.ox.ac.uk

†Contributed equally

Abstract

Background: The Investigation/Study/Assay (ISA) Metadata Framework is an established and widely used set of open-source community specifications and software tools for enabling discovery, exchange and publication of metadata from experiments in the life sciences. The original ISA software suite provided a set of user-facing Java tools for creating and manipulating the information structured in ISA-Tab – a now widely used tabular format. To make the ISA framework more accessible to machines and enable programmatic manipulation of experiment metadata, a JSON serialization ISA-JSON was developed.
Results: In this work, we present the ISA API, a Python library for the creation, editing, parsing, and validating of ISA-Tab and ISA-JSON formats by using a common data model engineered as Python object classes. We describe the ISA API feature set, early adopters and its growing user community. **Conclusions:** The ISA API provides users with rich programmatic metadata handling functionality to support automation, a common interface and an interoperable medium between the two ISA formats, as well as with other life science data formats required for depositing data in public databases.

Keywords

metadata, standards, reproducibility, open-source software, life science, API

Findings

Background

There are many data models and frameworks for describing entities and artefacts of scientific research. The life sciences have pioneered the development and application of ontologies, data standards, and minimum standards for reporting research results. More than ever, the importance of making scientific data FAIR (Findable, Accessible, Interoperable and Reusable) is at the forefront of discourse in the research community [1].

The ISA Metadata Framework, or simply ISA – so named after its constituent key concepts: 'Investigation' (the project context), 'Study' (a unit of research) and 'Assay' (analytical measurement), was born out of initial efforts to create an 'exchange network test bed' for a diverse set of digital resources in 'omics studies. These included public and proprietary databases, as well as free and open source software tools and commercially developed systems. ISA specifies a data model and standard serialization formats for describing experiments in the life sciences.

At the heart of the ISA is a general-purpose data model; an extensible, hierarchically structured model that enables the representation of studies employing one or a combination of technologies, focusing on the description of their experimental metadata (i.e. sample characteristics, technology and measurement types, and sample-to-data relationships).

ISA was originally conceptualized and specified as the ISA-Tab format [2,3] for describing life science experiments. Refinements to the underlying data model were later captured in a new JSON-based serialization format, ISA-JSON [4,5] that is specified using JSON schemas [6]. A data model was then abstracted from both the tabular and JSON formats in order to formalize the core concepts and their relationships to one another. The ISA data model is formed around three core concepts: *Investigations*, *Studies* and *Assays* (Figure 1).

[Figure 1 to appear here]

Figure 1. An overview of the ISA data model showing its key constituent classes and their relationships with one another. The model is structured around the concepts of investigation, study and assay (in red). Other model elements exist to qualify these core elements (in green), for example relating investigations and sub-studies with relevant contact persons or related publications; or information about the study design used and any experimental protocols implemented. Experimental workflows are modelled as sequences of process events with inputs and outputs that correspond to biological materials and data objects (in blue). Values can be made explicit by using term annotations or unit declarations that reference published ontologies (in orange).

Assays refer to specific tests performed on sample material taken from a subject, or performed on a whole subject, that produces some kind of qualitative or quantitative measurements, which correspond to response variables. Assay metadata describes sample-to-data relationships, grouped by analytical measurement types (e.g. metabolite profiling, DNA profiling) and technologies (e.g. Mass Spectrometry or Nuclear Magnetic Resonance, if the measurement type is metabolite profiling).

Study objects hold metadata about the subject(s) under study, including properties about the individual subject(s), any treatments (biological or statistical) applied, and the provenance of sample material back to the original source. Experimental factors relating to the subjects and samples are stored here, allowing the definition of specific study groups in relation to the study independent variables.

Investigations contain all the information needed to understand the overall goals and means used in an experiment and are used as high-level objects to group multiple related Study objects. For each Investigation, there may be one or more Studies associated with it; for each Study there may be one or more Assays.

The ISA data model allows us to find scientific experiments of interest by having high-level metadata about the experiments such as what the studies are about and more concretely with descriptors of the Study Design type (a classifier for the study based on the overall study design, e.g. crossover design or parallel group design) and study factors used, e.g. independent variables manipulated by the experimentalist in the study. Furthermore,

experiments can be searched by Assay types, on the type of measurements being carried out in the study and the technologies used to perform the measurements.

A key feature in the ISA data model is the use of ontology terms for certain fields in the metadata, supported with the Ontology Annotation and Ontology Source classes. Where appropriate, data model objects can be qualified with ontology terms (Ontology Annotations) that are linked to a declared description of the source of the terms (Ontology Sources). Supporting software tools can therefore populate such annotations from established terminology services such as the NCBI BioPortal [7,8] or the EMBL-EBI Ontology Lookup Service [9,10]. By providing support for discretely identified terms, we enable the ability to search across ISA objects stored in different experiments.

The data model enables experimentalists to describe metadata relating to the provenance of samples and data. This provenance is modelled in the form of directed acyclic graphs (i.e. vertices connected together by edges, but without any loops/cyclic dependencies), which describe the workflow undertaken from subject to sample to the production of data, including associated data acquisition files, analysis data matrices and discoveries being preserved in reusable form.

Full details about the model, as well as specifications for ISA-Tab and ISA-JSON are available from [11].

The supporting open-source ISA tools form a software suite designed to manage the experimental metadata using the aforementioned ISA formats, and more specifically to: (i) customise, describe and validate, following community reporting standards (with the ISAconfigurator, ISAcreator and ISAvalidator components [12] or OntoMaton [13]), (ii) store and browse, locally or remotely (BioInvestigation Index [14] and the ISAexplorer [15]); (iii) submit to public repositories (ISAconverter [12]); (iv) analyse with existing tools (Risa) [16]; and (v) publish data alongside the article. The adoption of these Java-based and web-based software tools has helped grow the ISA community of users, characterised by those listed in the ISA Commons [17].

As the original ISA software suite does not provide a programmatic interface, we have developed the ISA API to position the ISA framework as an interoperable and open platform [18]. We also recognized a trend of growing enthusiasm by end users for writing software code. This trend is perhaps most evident in statistical programming platforms such as R, Python and MatLab, and also greatly helped with the uptake of interactive programming environments such as Project Jupyter [19].

The aim of the ISA API is to reproduce much of the user-facing functionality afforded by the ISA software suite and to additionally enable interoperation between software systems that produce and consume ISA formats and other machine-readable data formats, as illustrated in Figure 2. The ISA API is written in Python, which has a high uptake by non-programmers and a rich open source community ecosystem of supporting software packages, including many statistical and bioinformatics ones. The software code is available on GitHub [20] under the Common Public Attribution License Version 1.0 (CPAL-1.0) and the software package is available via the Python Package Index (PyPI) [21] and Bioconda [22] under the moniker: `isatools` [23,24].

[Figure 2 to appear here]

Figure 2. ISA API and its interactions with other software ecosystems and data formats. Apart from running in the Python interpreter, the ISA API can also be accessed through the iPython, Jupyter and Docker. It also supports interoperation with other systems through standardised machine-readable data formats.

Related work

Developed in early 2011, the `Bio-Parser-ISATab` project [25] is, to the best of our knowledge, the first effort to create an ISA-Tab parser outside of the ISA tools ecosystem for programmatic manipulation of ISA metadata. While `Bio-Parser-ISATab` is written in the Perl programming language, the first Python project to implement an ISA-Tab parser with scripts found in the `biopy-isatab` project [26] was developed as part of an early version of `bcbio` [27] later in 2011. Both efforts focused only on reading ISA-Tab.

Recent efforts to create ISA programming libraries have focused on reading and writing of ISA-Tab files: AltamISA [28] was developed in Python with the aim for strict validation and error handling when handling ISA-Tab files; and a Java library, isa4j [29], has been developed for high-performance writing of ISA-Tab metadata.

ISA API provides a reference implementation of ISA-Tab support and additionally supports other formats and features as described in the following section.

Implementation

The ISA API is written in the Python 3 programming language, and therefore part of the wider Python ecosystem of software tooling which is very popular in the bioinformatics and data science communities [30]. The ISA API can immediately be imported and used alongside other Python packages in custom Python programs, or it can be used interactively via iPython's interactive shell [31] and in Jupyter Notebook web applications. It can be used in cloud infrastructure that supports Docker through the container image `isatools/isatools` published in DockerHub. The ISA API can be also exposed through RESTful APIs as demonstrated by the MetaboLights Labs Web service interface and the MetaboLights Online Editor Web service [32].

ISA API features

The ISA API provides a range of features that are summarized in this section.

Support for all ISA formats

The ISA API provides the solution to simplify access and generation of ISA metadata now that the ISA framework supports more than one on-disk data format – i.e., ISA-Tab and ISA-JSON. By using the ISA API, software can support full read, write and validation transparently on all current and future ISA formats without any additional complexity.

The ISA-Tab and ISA-JSON document validators included in the ISA API provide reference implementations by which to also validate documents generated by other systems that claim ISA compatibility [17].

Support for other bioinformatics formats

The ISA API also supports related domain formats used by bioinformatics communities and which are usually technology-centric (e.g. mass spectrometry, sequencing, DNA microarrays). This feature is a consequence of the ISA framework's support for multi-modality datasets (e.g. multi-omics datasets). The ISA API supports the import and export of MAGE-TAB [33,34] and SampleTab [35,36], import of metadata from mzML [37,38] and nmrML [39,40], and exports to SRA-XML [41,42] and Workflow4metabolomics [43].

Export metadata for submission to public repositories

The ability to export metadata to a range of formats means that software using the ISA API can prepare metadata submissions to public data repositories. The ISA API supports export of ISA metadata to submission-ready formats for public repositories such as the EMBL-EBI ArrayExpress [44,45], European Nucleotide Archive [46,47]s, and MetaboLights [48,49]. Support for submission to additional databases will be developed in future.

Multiple modes of handling ISA metadata

The ISA API is designed to support multiple modes of reading and writing ISA. Metadata reading and writing can be done natively with ISA-Tab folders, ISArchive zip files, and ISA-JSON files or strings. ISA metadata can be manipulated after loading using an object-oriented Python class model. Direct conversion between ISA formats is also supported.

Extensible and coherent object-oriented class model

Metadata objects are represented in the ISA API using an object-oriented class model, providing a format-agnostic in-memory representation. This in-memory model is based on

the ISA Abstract Model, which was extracted from the original ISA-Tab specification to formalize the concepts used in the ISA framework. It describes the steps in an experimental workflow as a directed acyclic graph (DAG), which in the ISA API is expressed naturally as connected Python class objects. For example, metadata relating to the assay description is held in an `Assay` object that is in-turn contained by a `Study` object and its relevant attributes, which is finally contained by an `Investigation` object – thus reflecting the hierarchical nature of the ISA framework concepts. All ISA concepts are modelled as classes at a granular level giving software developers full control to extend the API's capabilities and build new features. Details of the class model used in the ISA API are published in the ISA Model and Serialization Specifications 1.0 [4].

This functionally rich object-based representation completely avoids many of the complexities associated with handling ISA metadata as tables – e.g., joining data from the sample and assay tables, handling multiple table rows per edge of the DAG. While in memory, the experimental metadata can be programmatically processed and manipulated. Moreover, the ISA API's object-based representation is a powerful decoupling tool. First, it decouples the client application from the specific ISA file format being handled. Second, it decouples the input and output metadata formats. When metadata is read, the format-specific parser creates the standardized in-memory representation, which can be edited and manipulated; when writing, a format-specific serialization routine traverses the objects to extract the data structure according to the target ISA format. Writing the ISA API object model to ISA-Tab requires converting experimental workflow graphs to tabular formats. Such work is described in the `graph2tab` library by Brandizi et al [50]. However, in that work, the authors only describe cases of unidirectional transformations from graphs to tables; ISA API implements bidirectional transformations between each of the two ISA formats and ISA content expressed as Python objects that includes a representation of DAGs. ISA content can also be authored by directly creating Python objects, a brief example of which is shown in Figures 3 and 4.

[Figure 3 to appear here]

Figure 3. Example of a Python script using the ISA API's class model objects to programmatically construct metadata about a study and serialize it to ISA-Tab. This script first creates the Investigation and Study structures that store general metadata about the experiment being described. Next, a source material object is created, and three sample materials. These are connected as inputs and outputs respectively to a sample collection process, forming a workflow from source to samples. This is then added to the study's 'process sequence': a container for experimental process event descriptions. Finally, the composition of the model objects is serialized as ISA-Tab to the standard output. Scripts such as these can form part of a larger Python software program, or executed directly from the command-line, to automate the construction of ISA metadata descriptors.

[Figure 4 to appear here]

Figure 4. Example Jupyter Notebook using the ISA API to use ISA class objects to programmatically construct metadata about a study, using similar code to that shown in Figure 3. Being Python-based, ISA API can integrate with any notebook environment that supports Python kernels including Jupyter Notebook, JupyterLab and JupyterHub, and proprietary notebook environments such as Google Colab [51]*, Microsoft Azure Notebooks* [52]*, and Amazon's SageMaker* [53]*. A set of Jupyter notebooks detailing how to use ISA-API key functionalities is available on GitHub* [54]*.*

Querying over ISA content

Common use cases for ISA metadata involve gathering samples and data files produced by assays that satisfy certain selection criteria. The criteria are objects based on sample characteristics, processing parameters, and study factor combinations (treatment groups). When ISA metadata is loaded as in-memory model objects, this representation can be queried using native Python code, for example by filtering using list comprehensions with conditional logic. The ISA API also provides helper functions, in the `isatools.utils` package, to query and fix possible encoding problems in ISA content. For example, in ISA-Tab files, a common issue is when ISA content is encoded in such a way that some branches in experimental graphs converge unexpectedly which represents the intended workflow incorrectly. The ISA API provides specific functions to detect such anomalies (in the `detect_isatab_process_pooling` function) and to fix them (in the

`insert_distinct_parameter` function). Details of how to query over ISA content can be found in the ISA API online documentation [55].

Semantic markup

An important feature that is embedded into the class model is the support for ontology annotation objects to better qualify metadata elements, contributing to making them Findable, Accessible, Interoperable, and Reusable (FAIR) [56]. The ISA API implements the `OntologyAnnotation` class that is used extensively throughout. To express quantitative values, units are implemented with the `Unit` class, which itself can be qualified with an `OntologyAnnotation` object. Populating annotations is aided by a network package in the ISA API for connecting to and querying the Ontology Lookup Service (OLS) [9]. JSON-LD context files complementing the ISA-JSON schemas mapping into schema.org [57] or OBO Foundry based resources [58] are also available.

Assisted curation of study metadata

Studies published using ISA formats are increasingly commonplace, many of which are produced in ISA-Tab using the ISAcreator (for example, NASA GeneLab [59,60] and MetaboLights [48]), and many others produced either by-hand (using a spreadsheet or text editor) or output by third-party systems (for example, COPO [61], FAIRDOM/SEEK [62,63] and SCDE [64,65]). There are sometimes cases of invalid ISA-formatted content appearing in public databases or reported via bug reports to the ISA development team. To help with this, the ISA API includes features to assist with metadata curation of ISA-formatted studies. Beyond using the validators contained in the API, additional features include checking for possible structural problems in the DAGs that describe the provenance of samples and data files (for example, where converging edges are detected but are not expected) and correcting them; re-situating incorrectly placed attributes (e.g. recasting incorrectly labelled `Factors` as `Parameters` or `Characteristics`); and batch fixing such issues. This work has resulted in the creation of curation functions, which have been applied to the content of

the MetaboLights database as a means to improve the quality and consistency of ISA archives. These validators also form the basis of the current MetaboLights extensive online validations coupled to their submission infrastructure to validate the metadata for missing values, consistency and sufficiency.

Assisted creation of study metadata

The ISA API *create* module contains a set of functions and methods exploiting study design information to bootstrap the creation of ISA content [66]. Initially created to support factorial designs, this component is currently being extended to provide support for longitudinal studies and repeated treatment designs. This work in progress will be refined in future versions of the ISA API.

Documentation

Extensive documentation is available as a Jupyter Book that includes examples presenting the various capabilities afforded by the ISA API [55]. The Jupyter Book includes information about how to install and use the `isatools` Python package, details about the ISA data model, and source code examples of using ISA API as Python scripts and Jupyter notebooks. For more information about the features listed here, interested readers are invited to view the ISA tools API documentation for detailed and up-to-date information.

Early adopters

The ISA API has a number of early adopters that demonstrate its value as an open platform to the ISA framework. The following section details how these groups rely on our python library in their data management tasks.

The MetaboLights metabolomics database [48] is using the ISA API to load, edit, and save ISA-Tab as part of its next-generation data curation interface. MetaboLights has redeveloped its user and data curator interface building on the latest Web technologies and using the ISA API to convert from ISA-Tab to ISA-JSON for further processing. The developers take a

service-oriented approach exposing various features for editing and updating ISA content through a RESTful API [32].

The PhenoMeNal (Phenome and Metabolome aNalysis) e-infrastructure for molecular phenotype data analysis has developed a set of containerised microservices (using Docker) that utilise ISA API's converters, validators, and study creation features [67]. The PhenoMenal Virtual Research Environment (VRE) is based on the Galaxy workflow platform [45] through its integration with Kubernetes [68]. Through PhenoMeNal, a suite of tools has been developed to integrate the ISA API with Galaxy [69]. The tools have been published as a Galaxy workflow since the Dalcotidine release of PhenoMeNal to demonstrate a study metadata preparation and pre-submission pipeline to the MetaboLights database (see Figure 5).

[Figure 5 to appear here]

Figure 5. The ISA Create-Validate-Upload workflow, published as part of the PhenoMeNal platform Dalcotidine release. The workflow takes a user-configured study plan and creates an ISA-Tab template ready for the experimentalist to use in their study. The ISA-Tab then goes through two paths of processing: (1) a summary of study factors according to the study design is extracted and then visualized as a parallel sets plot; and (2) the ISA-Tab is validated, and if valid a pre-submission request is made by uploading the ISA-Tab to MetaboLights Labs. A preparatory study accession ID is then issued by MetaboLights if accepted. Parts of the workflow that directly use the ISA API are highlighted in green along with the packages used.

The Galaxy workflow platform includes native support for ISA formats by using the ISA API in its datatype implementations of ISA-Tab and ISA-JSON, paving the way for Galaxy tools to consume and produce ISA content as first-class Galaxy datasets.

The Collaborative Open Omics (COPO) platform [70] supports ISA-JSON as one of its metadata formats and uses ISA API's SRA exporter to allow COPO users to publish studies directly to the European Nucleotide Archive. COPO configures its back end and front end for brokering various data types, including omics data, through consuming ISA configurations. The ISA configurations inform how COPO should present the data preparation wizard to the user, and how it stores it in its database in a variation of ISA-JSON. COPO stores study

metadata as JSON where it conforms to the ISA-JSON schemas and adds extra metadata relating to its operation within the COPO database. By supporting ISA-JSON, COPO lends itself to easily leveraging the ISA API to export data to formats supported by the API.

Still within the plant science community, a conversion from BrAPI [71] to ISA has been developed along with MIAPPE compliant ISA configurations [72,73]. The functionality will be integrated along with other community conversion components in future releases of the ISA API.

A stable and growing user community

Since its first release, ISA API has grown its user base steadily with active contributions from the community as an open source project as evidenced by bug reports, feature requests, and code contributions. While it is difficult to measure the uptake of free software, ISA API has been available since 2016 via PyPI, which collects download statistics for all packages it hosts [74]. There are documented drawbacks to using these PyPI download statistics, such as historical data issues and systemic irregularities such as caching of downloads that can cause undercounting, so we view these statistics as indicative of how the ISA API project has progressed rather than as an accurate measure. These statistics also do not include installations directly from the source code repository on GitHub or from Bioconda.

[Figure 6a to appear here] [Figure 6b to appear here]

Figure 6. Download statistics the `isatools` Python package from PyPI from 21 February 2016 (first release of ISA API, v0.1) up to 24 October 2020 (after release v0.11). Note that the total number of downloads for 2020 was incomplete at the time the data was collated. Sub-figure (6a) shows a bar chart depicting the annual total downloads of `isatools`. Sub-figure (6b) shows a line chart depicting the monthly total number of downloads of `isatools` with major releases of the ISA API indicated with vertical dashed lines.

After its initial release year, we observed a maintained and slight year-on-year growth from 2017 to 2019 of around 27,000 annual downloads via PyPI (Figure 6), which we believe demonstrates that the ISA API has built up a stable and active user base.

When looking at a more granular picture of the download statistics for the `isatools` package, on a month-by-month basis, we observe spikes in downloads on PyPI after each major release. We believe this indicates that the ISA API user community continues to be active and is using the platform in production systems by migrating to its newest updates soon after they are made available.

Conclusions

The development of the ISA API represents a major step forward in making the ISA framework open and interoperable, enabling better handling of experimental metadata, and supporting a diverse user community. By providing a programming interface, rather than a graphical user interface, the ISA API provides a platform for simplified and consistent integration of the ISA framework in new and existing software, promoting the production of structured metadata by agents and the development of data management solutions that can be FAIR by design. The availability of an ISA implementation that is easily used by other software also reduces the likelihood of independent implementations that may not strictly adhere to the ISA format specifications. The object-oriented class model provides a format-agnostic interface for client software allowing software that uses the ISA API to automatically support all ISA file formats, while also providing a common basis on which to build data format parsers and serializers more easily. Should external implementations of the ISA formats be required, by providing reference implementations of ISA-Tab and ISA-JSON parsers, serializers and validators, software developers have the infrastructure in which to extend the API or build interoperation of ISA with other software systems. As Python code, it can also easily integrate into existing bioinformatics and data science platforms such as Galaxy and Jupyter. Importantly, the object-oriented DAG model presented by the ISA API offers a more intuitive way to reason about and process study metadata than as ISA-Tab tables, simplifying the work of developers and enabling the development of richer metadata

processing applications. The ISA API is available as free and open-source software which will improve the dissemination and application of the ISA metadata framework.

Methods

Community-led requirements

The ISA metadata framework and its supporting tools were developed to support requirements of the life science research community and its requirements are elicited in collaboration with a wide range of stakeholders, such as those represented in the ISA Commons [17]. This approach continues with the development of the ISA API where, through direct research funding, the project was initially driven by requirements of metabolomics research (via the H2020 PhenoMeNal project [67]) and the plant sciences community (via the UK BBSRC COPO project [70]). Development has also been guided by collaboration workshops (for example [75] and [76]). As an open source project from its outset in 2015, the ISA API openly encourages grass-roots contributions via its GitHub project, where we receive continuous feedback, feature requests, and source code contributions from third parties.

Model-driven engineering

The ISA API was developed using a model-driven engineering (MDE) approach [77] that initially focused heavily on formalising the concepts that define the ISA metadata framework. This required close analysis of the only specification of ISA concepts available documented in the ISA-Tab 1.0 specification [2] and the de facto reference implementation of ISA-Tab in the Java source code of the ISA software suite [12], as well as critical discussions within the ISA Working Group. One of the early goals of the ISA API project was to implement support for a JSON serialization format, ISA-JSON [4]. A model specification was drafted as a set of

JSON schemas, the JSON community's response to implementing features reminiscent of XML schemas. This was then formalised as the first data agnostic specification of ISA.

From the ISA-JSON schemas, we then used the `warlock` Python package [78] to draft an object-oriented (OO) Python class model (via Python's dynamic object creation facility) that was highly synchronised with the JSON schemas. This OO class model was then implemented concretely (to provide object reflection/introspection that dynamic objects lack) to use as the primary representation of ISA metadata in Python. Data format parsers and serializers were then developed to load data into these Python objects at run-time. The ISA API implements support interoperation between formats such as ISA-Tab, ISA-JSON, SampleTab, MAGE-TAB and SRA-XML by using these ISA Python objects.

It is important to note that the ISA data model is not a model of ISA-Tab; rather, ISA-Tab is an implementation of the data model. The representation of ISA concepts in a tabular form is specific to the serialization format, where ISA is not about tables, rows and headers. ISA metadata is about the encapsulated semantics about the life science experiments being described. However, in developing the class model, ISA-Tab parser and serializer it was realised that some ISA-Tab concepts were required in the class model to support serialization.

For example, the ISA-Tab table file names are not strictly part of the ISA data model but we need to be able to specify these to support writing ISA-Tab files. When writing to ISA-JSON these file name attributes are ignored as the JSON implementation is more closely aligned to the model. Therefore, the ISA API's implementation of the model should be considered as only very closely aligned with the model specification rather than a perfect mapping.

Test-driven development

When developing new features and fixing bugs, the ISA API development team predominantly follows a test-driven development (TDD) approach [79]. This means that when new features are requested an automated unit test is implemented first to capture the

feature specification, then work is done to develop the new feature to satisfy the test. Similarly, when a bug is reported via the project's GitHub issue tracker, an automated regression test is implemented first to capture the problem specification before developing the bugfix. Automated unit tests are implemented using the `unittest` package and run with the `nose` and `tox` testing frameworks. Source code contributions to the GitHub project are automatically tested on multiple Python versions using the continuous integration service Travis CI.

ISA API's most recent release at time of writing (v0.12) uses 631 automated unit tests.

Download statistics

The download statistics for the `isatools` package hosted on PyPI shown in Figure 6 are collected by the Linehaul statistics daemon [80] that logs downloads of all Python packages on PyPI and pushes the data to a public dataset [74] on Google BigQuery [81]. The all-time statistics about the `isatools` package were retrieved from this public dataset on 24 October 2020 at 14:20 UTC+2 with the following SQL query via the BigQuery interface:

```
SELECT TIMESTAMP_TRUNC(timestamp, WEEK) week
, COUNT(*) downloads
FROM `the-psf.pypi.downloads*`
WHERE file.project='isatools'
GROUP BY week, file.version
ORDER BY week
```

The result of this query was downloaded as a CSV file containing a timestamp of the week-ending each weekly period of downloads, the `isatools` version number, and the total number of downloads for each period and version. This data was then aggregated by month and by year before being visualized using the Seaborn statistical data visualization package [82].

Data about the version release dates of ISA API were collected from the ISA API GitHub repository [20].

Availability of supporting data

The all-time weekly download statistics data for the `isatools` package on PyPI up to 24 October 2020 14:20 UTC+2 and the code used to plot the charts used in Figures 6a and 6b in this manuscript are available in a Code Ocean capsule [83]. The data and code is available under CC BY 4.0 and MIT License respectively.

Availability of source code and requirements

- Project name: ISA API
- Project home page: http://github.com/ISA-tools/isa-api/
- Operating system(s): Platform independent
- Programming language: Python
- Other requirements: Python 3.6+
- License: CPAL-1.0
- biotools: isa_api
- RRID: SCR_020980

Declarations

List of abbreviations

API: Application Programming Interface

BBSRC: Biotechnology and Biological Sciences Research Council

BrAPI: Breeding API

COPO: Collaborative Open Plant Omics

DAG: Directed Acyclic Graph

EMBL: European Molecular Biology Laboratory

EMBL-EBI: European Bioinformatics Institute

GUI: Graphical User Interface

H2020: Horizon 2020

ISA: Investigation, Study, Assay

ISA-JSON: ISA JavaScript Object Notation format

ISA-Tab: ISA Tabular format

JSON: JavaScript Object Notation

JSON-LD: JavaScript Object Notation for Linked Data

MAGE-TAB: MicroArray Gene Expression-Tabular format

MIAPPE: Minimum Information About a Plant Phenotyping Experiment

mzML: Mass spectrometry Markup Language

NASA: National Aeronautics and Space Administration

NERC: Natural Environment Research Council

NCBI: National Center for Biotechnology Information

nmrML: Nuclear magnetic resonance Markup Language

MDE: Model-driven engineering

OLS: Ontology Lookup Service

OO: Object-oriented

PhenoMeNal: Phenome and Metabolome aNalysis

PyPI: Python Package Index

REST: Representational state transfer

RDF: Resource Description Framework

SCDE: Stem Cell Discovery Engine

SQL: Structured Query Language

SRA-XML: Sequence Read Archive-eXtensible Markup Language

TDD: Test-driven development

VRE: Virtual Research Environment.

Consent for publication

Not applicable.

Competing Interests

All authors declare that they have no other competing interests.

Funding

This work has been supported in part by: European Commission Horizon 2020 programme *PhenoMeNal* project (grant agreement no. EC654241); UK BBSRC *COPO* project (Bioinformatics and Biological Resources Fund (BBR) grant: BB/L021390/1 [BB/L024055/1, BB/L024071/1, BB/L024101/1]); UK BBSRC *Establishing common standards and curation practices: towards real world biosharing* grant (BB/J020265/1); UK BBSRC *Sharing of metabolomics data and their analyses as Galaxy workflows through a UK-China collaboration* grant (BB/M027635/1); and a UK NERC CASE Ph.D. studentship in collaboration with GigaScience: (NE/L002493/1).

A.G-B., K.H., M.I., D.J., M.L., T.N.L., P.M., L.P., P.R-S, P.R., S-A.S., C.S. and R.J.M.W. received funding from the EC H2020 PhenoMeNal project grant. A.E., R.P.D., A.G-B., D.J., P.R-S, S-A.S., and F.S. received funding from the UK BBSRC COPO project grant. T.N.L. received funding from the NERC CASE Ph.D. studentship.

Author's Contributions

Conceptualization: P.R-S, S-A.S.; software-lead: D.J.; software-supporting: M.I., P.R-S, A.G-B, K.C., A.E., K.H., M.L., T.N.L., A.M., P.M., V.C.N., L.P., P.R., F.S., R.J.M.W.; investigation-usage: D.J.; visualization-usage: D.J.; writing-original draft: D.J.; writing-review and editing: D.J., P.R-S., K.C., R.P.D., A.E., A.G-B, K.H., M.I., M.L., T.N.L., P.M., V.C.N., C.O., L.P., P.R., F.S., C.S., R.J.M.W., S-A.S.; funding acquisition: R.P.D., C.O., S-A.S, C.S.

Acknowledgements

We thank members of the ISA Working Group, ISA Commons, H2020 PhenoMeNal project, UK BBSRC COPO project, attendees of the 2016 “Hack-the-Spec - ISA as a FAIR research object” workshop hosted by the Oxford e-Research Centre, UK, attendees of the 2016 “China-UK Data Dissemination in Metabolomics (CUDDEL)” workshop hosted by GigaScience, Hong Kong, and the EMBL-EBI MetaboLights team for guidance and feedback on the development of the ISA API.

References

1. McQuilton P, Batista D, Beyan O, Granell R, Coles S, Izzo M, et al. Helping the consumers and producers of standards, repositories and policies to enable FAIR Data. Data Intell. 2019; doi:10.1162/dint_a_00037.
2. Rocca-Serra P, Sansone S-A, Brandizi M. Specification documentation: ISA-TAB 1.0. Zenodo. 2009; doi:10.5281/zenodo.161355.
3. FAIRsharing.org: ISA-Tab; Investigation Study Assay Tabular; DOI: https://doi.org/10.25504/FAIRsharing.53gp75; Last edited: Feb. 8, 2021, 1:22 p.m.; Last accessed: Mar 03 2021 7:54 a.m.
4. Sansone S-A, Rocca-Serra P, Gonzalez-Beltran A, Johnson D, ISA Community. ISA model and serialization specifications 1.0. Zenodo. 2016; doi:10.5281/zenodo.163640
5. FAIRsharing.org: ISA-JSON; Investigation Study Assay JSON; DOI: https://doi.org/10.25504/FAIRsharing.yhLgTV; Last edited: March 10, 2021, 1:08 p.m.; Last accessed: Mar 12 2021 10:33 a.m.
6. Pezoa F, Reutter JL, Suarez F, Ugarte M, Vrgoč D. Foundations of JSON Schema. In: Bourdeau J, editor. Proceedings of the 25th International Conference on World Wide Web (WWW ‘16). New York: ACM; 2016. p. 263-273.
7. Whetzel PL, Noy NF, Shah NH, Alexander PR, Nyulas C, Tudorache T, et al.

BioPortal: enhanced functionality via new Web services from the National Center for Biomedical Ontology to access and use ontologies in software applications. Nucleic Acids Res. 2011; doi:10.1093/nar/gkr469.

8. FAIRsharing.org: BioPortal; BioPortal; DOI: https://doi.org/10.25504/FAIRsharing.4m97ah; Last edited: Dec. 16, 2020, 1:20 p.m.; Last accessed: Mar 03 2021 8:03 a.m.
9. Jupp S, Burdett T, Leroy C, Parkinson HE. A new ontology lookup service at EMBL-EBI. In: Malone J, Stevens R, Forsberg K, Splendiani Al, editors. Proceedings of the 8th International Conference on Semantic Web Applications and Tools for Life Sciences (SWAT4LS 2015). Aachen: CEUR-WS.org; 2015.
10. FAIRsharing.org: OLS; Ontology Lookup Service; DOI: https://doi.org/10.25504/FAIRsharing.Mkl9RR; Last edited: Jan. 25, 2021, 4:27 p.m.; Last accessed: Mar 03 2021 8:12 a.m.
11. ISA Model and Serialization Specifications. https://isa-specs.readthedocs.io/en/latest/isamodel.html. Accessed 9 Oct 2020.
12. Rocca-Serra P, Brandizi M, Maguire E, Sklyar N, Taylor C, Begley K, et al. ISA software suite: supporting standards-compliant experimental annotation and enabling curation at the community level. Bioinformatics. 2010. doi:10.1093/bioinformatics/btq415.
13. Maguire E, Gonzalez-Beltran A, Whetzel PL, Sansone S-A, Rocca-Serra P. OntoMaton: a BioPortal powered ontology widget for Google Spreadsheets. Bioinformatics. 2013;29:525-527.
14. BioInvestigation Index on GitHub. https://github.com/ISA-tools/BioInvIndex. Accessed 2 Mar 2021.
15. Gonzalez-Beltran A. ISA-explorer: A demo tool for discovering and exploring Scientific Data's ISA-tab metadata. http://blogs.nature.com/scientificdata/2015/12/17/isa-explorer/. Accessed 11 Nov 2020.

16. Gonzalez-Beltran A, Neumann S, Maguire E, Sansone S-A, Rocca-Serra P. The Risa R/Bioconductor package: integrative data analysis from experimental metadata and back again. BMC Bioinformatics. 2014; doi:10.1186/1471-2105-15-S1-S11.
17. ISA commons. https://www.isacommons.org/. (2018). Accessed 9 Oct 2020.
18. Eisenmann TR, Parker G, Van Alstyne M. Opening platforms: how, when and why? In: Gawer A, editor. Platforms, markets and innovation. Cheltenham: Edward Elgar Publishing; 2009. p. 131-162.
19. Kluyver T, Ragan-Kelley B, Pérez F, Granger BE, Bussonnier M, Frederic J, et al. Jupyter Notebooks - a publishing format for reproducible computational workflows. In: Proceedings of the 20th International Conference on Electronic Publishing (ELPUB 2016). Amsterdam: IOS Press; 2016. p. 87-90.
20. ISA-tools/isa-api on GitHub. https://github.com/ISA-tools/isa-api/. Accessed 9 Oct 2020.
21. The Python Package Index. https://pypi.org/. Accessed 29 Oct 2020.
22. Grüning B, Dale R, Sjödin A, Chapman BA, Rowe J, Tomkins-Tinch CH, et al. Bioconda: sustainable and comprehensive software distribution for the life sciences. Nat Methods.; 2018; doi:10.1038/s41592-018-0046-7.
23. ISA API (isatools) on PyPI. https://pypi.org/project/isatools/. Accessed 9 Oct 2020.
24. ISA API (isatools) on Bioconda. https://anaconda.org/bioconda/isatools/. Accessed 9 Oct 2020.
25. bobular/Bio-Parser-ISATab on GitHub. https://github.com/bobular/Bio-Parser-ISATab. Accessed 2 Mar 2021.
26. ISA-tools/biopy-isatab on GitHub. https://github.com/ISA-tools/biopy-isatab. Accessed 2 Mar 2021.
27. Brad Chapman, Rory Kirchner, Lorena Pantano, Sergey Naumenko, Matthias De Smet, Luca Beltrame, et al. bcbio/bcbio-nextgen: v1.2.7. Zenodo; 2021.
28. Kuhring M, Nieminen M, Kirwan J, Beule D, Holtgrewe M. AltamISA: a Python API for ISA-Tab files. J Open Source Softw. 2019; doi: 10.21105/joss.01610.

29. Psaroudakis D, Liu F, König P, Scholz U, Junker A, Lange M, et al.. isa4j: a scalable Java library for creating ISA-Tab metadata. F1000Research. 2020; doi: 10.12688/f1000research.27188.1.

30. Russell PH, Johnson RL, Ananthan S, Harnke B, Carlson NE. A large-scale analysis of bioinformatics code on GitHub. PLoS ONE. 2018; doi:10.1371/journal.pone.0205898.

31. Pérez F, Granger BE. IPython: a system for interactive scientific computing. Comput Sci Eng. 2007:9:21-29.

32. MetaboLights RESTful WebService API specification. https://www.ebi.ac.uk/metabolights/ws/api/spec.html. Accessed 9 Oct 2020.

33. Rayner TF, Rocca-Serra P, Spellman PT, Causton HC, Farne A, Holloway E, et al. A simple spreadsheet-based, MIAME-supportive format for microarray data: MAGE-TAB. BMC Bioinformatics. 2006;7:489.

34. FAIRsharing.org: MAGE-TAB; MicroArray Gene Expression Tabular Format; DOI: https://doi.org/10.25504/FAIRsharing.ak8p5g; Last edited: Jan. 8, 2019, 1:40 p.m.; Last accessed: Mar 03 2021 8:26 a.m.

35. Courtot M, Cherubin L, Faulconbridge A, Vaughan D, Green M, Richardson D, et al. BioSamples database: an updated sample metadata hub. Nucleic Acids Res. 2019;47:D1172–D1178.

36. FAIRsharing.org: SampleTab; Sample Tabular Format; DOI: https://doi.org/10.25504/FAIRsharing.hgnk8v; Last edited: March 9, 2020, 10:30 a.m.; Last accessed: Mar 03 2021 8:28 a.m

37. Martens L, Chambers M, Sturm M, Kessner D, Levander F, Shofstahl J, et al. mzML- a community standard for mass spectrometry data. Mol Cell Proteomics. 2011;10.

38. FAIRsharing.org: mzML; mz Markup Language; DOI: https://doi.org/10.25504/FAIRsharing.26dmba; Last edited: Jan. 8, 2019, 1:27 p.m.; Last accessed: Mar 03 2021 8:30 a.m.

39. Schober D, Jacob D, Wilson M, Cruz JA, Marcu A, Grant JR, et al. nmrML: a

community supported open data standard for the description, storage, and exchange of NMR data. Anal Chem. 2018; doi:10.1021/acs.analchem.7b02795.

40. FAIRsharing.org: NMR-ML; Nuclear Magnetic Resonance Markup Language; DOI: https://doi.org/10.25504/FAIRsharing.es03fk; Last edited: Jan. 8, 2019, 1:31 p.m.; Last accessed: Mar 03 2021 8:31 a.m.
41. Kodama Y, Shumway M, Leinonen R. The Sequence Read Archive: explosive growth of sequencing data. Nucleic Acids Res. 2012;40:D54–D56 .
42. FAIRsharing.org: SRA-XML; Short Read Archive eXtensible Markup Language; DOI: https://doi.org/10.25504/FAIRsharing.q72e3w; Last edited: Feb. 11, 2020, 9:01 p.m.; Last accessed: Mar 03 2021 8:34 a.m.
43. Giacomoni F, Le Corguillé G, Monsoor M, Landi M, Pericard P, Pétéra M, et al. Workflow4Metabolomics: a collaborative research infrastructure for computational metabolomics. Bioinformatics. 2015; doi:10.1093/bioinformatics/btu813.
44. Athar A, Füllgrabe A, George N, Iqbal H, Huerta L, Ali A, et al. ArrayExpress update–from bulk to single-cell expression data. Nucleic Acids Res. 2019; 47:D711–D715.
45. FAIRsharing.org: ArrayExpress; ArrayExpress; DOI: https://doi.org/10.25504/FAIRsharing.6k0kwd; Last edited: Feb. 8, 2021, 8:54 p.m.; Last accessed: Mar 03 2021 8:35 a.m.
46. Amid C, Alako BT, Balavenkataraman Kadhirvelu V, Burdett T, Burgin J, Fan J, et al. The European Nucleotide Archive in 2019. Nucleic Acids Res. 2020;48:D70–D76.
47. FAIRsharing.org: ENA; European Nucleotide Archive; DOI: https://doi.org/10.25504/FAIRsharing.dj8nt8; Last edited: Feb. 23, 2021, 3:55 p.m.; Last accessed: Mar 03 2021 8:37 a.m.
48. Haug K, Cochrane K, Nainala VC, Williams M, Chang J, Jayaseelan KV, et al. MetaboLights: a resource evolving in response to the needs of its scientific community. Nucleic Acids Res. 2020;48:D440–D444.
49. FAIRsharing.org: MTBLS; MetaboLights; DOI: https://doi.org/10.25504/FAIRsharing.kkdpxe; Last edited: Feb. 4, 2021, 2:47 p.m.;

Last accessed: Mar 03 2021 8:39 a.m.

50. Brandizi M, Kurbatova N, Sarkans U, Rocca-Serra P. graph2tab, a library to convert experimental workflow graphs into tabular formats. Bioinformatics. Oxford University Press; 28:1665–16672012;
51. Google Colaboratory. https://colab.research.google.com/. Accessed 22 Oct 2020.
52. Microsoft Azure Notebooks. https://notebooks.azure.com/. Accessed 22 Oct 2020.
53. Amazon SageMaker. https://aws.amazon.com/sagemaker/. Accessed 22 Oct 2020.
54. Example Jupyter notebooks using the ISA-API on GitHub. https://github.com/ISA-tools/isatools-notebooks/. Accessed 28 Oct 2020.
55. The ISA cookbook. https://isa-tools.org/isa-api/content/index.html. Accessed 2 Mar 2021.
56. Wilkinson MD, Dumontier M, Aalbersberg IjJ, Appleton G, Axton M, Baak A, et al. The FAIR Guiding Principles for scientific data management and stewardship. Sci Data. 2016;3:1-9.
57. Guha RV, Brickley D, MacBeth S. Schema.org: Evolution of Structured Data on the Web: Big data makes common schemas even more necessary. ACM Queue. 2015; doi:10.1145/2857274.2857276.
58. Smith B, Ashburner M, Rosse C, Bard J, Bug W, Ceusters W, et al. The OBO Foundry: coordinated evolution of ontologies to support biomedical data integration. Nat Biotechnol. 2007; doi:10.1038/nbt1346.
59. Ray S, Gebre S, Fogle H, Berrios DC, Tran PB, Galazka JM, et al. GeneLab: Omics database for spaceflight experiments. Bioinformatics. 2019;35:1753–1759.
60. FAIRsharing.org: genelab; NASA GeneLab; DOI: https://doi.org/10.25504/FAIRsharing.64mr5a; Last edited: Feb. 5, 2021, 12:42 p.m.; Last accessed: Mar 03 2021 8:43 a.m
61. Shaw F, Etuk A, Gonzalez-Beltran A, Rocca-Serra P, Kersey PJ, Bastow R, et al. COPO-Linked Open Infrastructure for Plant Data. In: Malone J, Stevens R, Forsberg K, Splendiani Al, editors. Proceedings of the 8th International Conference on

Semantic Web Applications and Tools for Life Sciences (SWAT4LS 2015). Aachen: CEUR-WS.org; 2015.

62. Wolstencroft K, Owen S, Krebs O, Nguyen Q, Stanford NJ, Golebiewski M, et al. SEEK: a systems biology data and model management platform. BMC Syst Biol. 2015;9:1-12.

63. FAIRsharing.org: FAIRDOMHub; FAIRDOMHub; DOI: https://doi.org/10.25504/FAIRsharing.nnvcr9; Last edited: Aug. 1, 2020, 7:24 p.m.; Last accessed: Mar 03 2021 8:47 a.m.

64. Ho Sui SJ, Begley K, Reilly D, Chapman B, McGovern R, Rocca-Serra P, et al. The Stem Cell Discovery Engine: an integrated repository and analysis system for cancer stem cell comparisons. Nucleic Acids Res. 2012; doi:10.1093/nar/gkr1051.

65. FAIRsharing.org: SCDE; Stem Cell Discovery Engine; DOI: https://doi.org/10.25504/FAIRsharing.490xfb; Last edited: Feb. 8, 2021, 8:54 p.m.; Last accessed: Mar 03 2021 8:49 a.m.

66. Rocca-Serra P, Johnson D, Weber RJM, Pireddu L, Roger P, Gonzalez-Beltran A, et al. ISAcreate Galaxy tool for prospective data management with ISA format support - application to metabolomics datasets (poster). F1000Research. 2018; doi:10.7490/f1000research.1115757.1.

67. Peters K, Bradbury J, Bergmann S, Capuccini M, Cascante M, de Atauri P, et al. PhenoMeNal: processing and analysis of metabolomics data in the cloud. Gigascience. 2019;8:giy149.

68. Moreno P, Pireddu L, Roger P, Goonasekera N, Afgan E, Van Den Beek M, et al. Galaxy-Kubernetes integration: scaling bioinformatics workflows in the cloud. bioRxiv. 2018;bioRxiv.488643.

69. ISA Galaxy tools, tours, and other enhancements on GitHub. https://github.com/ISA-tools/isatools-galaxy/. Accessed 9 Oct 2020.

70. Shaw F, Etuk A, Minotto A, Gonzalez-Beltran A, Johnson D, Rocca-Serra P, et al. COPO: a metadata platform for brokering FAIR data in the life sciences.

F1000Research. 2020; doi:10.12688/f1000research.23889.1.

71. Selby P, Abbeloos R, Backlund JE, Basterrechea Salido M, Bauchet G, Benites-Alfaro OE, et al. BrAPI—an application programming interface for plant breeding applications. Bioinformatics. 2019; doi:10.1093/bioinformatics/btz190.
72. Ćwiek-Kupczyńska H, Altmann T, Arend D, Arnaud E, Chen D, Cornut G, et al. Measures for interoperability of phenotypic data: minimum information requirements and formatting. Plant Methods. 2016; doi:10.1186/s13007-016-0144-4.
73. FAIRsharing.org: MIAPPE; Minimum Information about Plant Phenotyping Experiment; DOI: https://doi.org/10.25504/FAIRsharing.nd9ce9; Last edited: Feb. 7, 2021, 9:09 p.m.; Last accessed: Mar 03 2021 8:54 a.m.
74. Analyzing PyPI package downloads - Python Packaging User Guide. https://packaging.python.org/guides/analyzing-pypi-package-downloads/. Accessed 28 Oct 2020.
75. ELIXIR-NL represented at ELIXIR UK's "ISA as a FAIR research object" event. https://www.dtls.nl/2015/09/16/defining-standards-to-describe-experiments-as-part-of-good-research-practices-the-isa-standard/. Accessed 2 Mar 2021.
76. CUDDELing up to metabolomics in Hong Kong. http://gigasciencejournal.com/blog/cuddeling-up-to-metabolomics-in-hong-kong/. Accessed 2 Mar 2021.
77. Schmidt DC. Model-Driven Engineering. IEEE Computer. 2006; doi: 10.1109/MC.2006.58.
78. bcwaldon/warlock on GitHub. https://github.com/bcwaldon/warlock. Accessed 2 Mar 2021.
79. Williams L, Maximilien EM, Vouk M. Test-driven development as a defect-reduction practice. 14th Int Symp Softw Reliab Eng 2003 ISSRE 2003.
80. The Linehaul Statistics Daemon. https://github.com/pypa/linehaul/. Accessed 28 Oct 2020.
81. BigQuery: Cloud Data Warehouse. https://cloud.google.com/bigquery/. Accessed 28

Oct 2020.

82. Michael Waskom, Olga Botvinnik, Maoz Gelbart, Joel Ostblom, Paul Hobson, Saulius Lukauskas, et al. mwaskom/seaborn: v0.11.0. Zenodo. 2020; doi:10.5281/zenodo.4019146

83. Johnson D. Code for the ISA API download statistics visualizations in “ISA API: An open platform for interoperable life science experimental metadata” [Source Code]. Code Ocean. 2020. https://doi.org/10.24433/CO.8813991.v1

Author information

Full name	Email address	Institution	ORCID
David Johnson	david.johnson@im.uu.se	Oxford e-Research Centre, Department of Engineering Science, University of Oxford, 7 Keble Road, OX1 3QG, Oxford, United Kingdom;<br><br>Department of Informatics and Media, Uppsala University, Box 513, 751 20 Uppsala, Sweden	0000-0002-2323-6847
Keeva Cochrane	keeva@ebi.ac.uk	European Molecular Biology Laboratory, European Bioinformatics Institute (EMBL-EBI), Wellcome Genome Campus, Hinxton, Cambridge CB10 1SD, United Kingdom	0000-0001-7961-0474
Robert P. Davey	robert.davey@earlham.ac.uk	Earlham Institute, Norwich Research Park, Norwich NR4 7UZ, United Kingdom	0000-0002-5589-7754
Anthony Etuk	tonietuk@gmail.com	Earlham Institute, Norwich Research Park, Norwich NR4 7UZ, United Kingdom	0000-0001-8320-660X
Alejandra Gonzalez-Beltran	alejandra.gonzalez-beltran@stfc.ac.uk	Oxford e-Research Centre, Department of Engineering Science, University of Oxford, 7 Keble Road, OX1 3QG, Oxford, United Kingdom;<br><br>Science and Technology Facilities Council, Scientific Computing Department, Rutherford Appleton Laboratory, Harwell Campus, Didcot, OX11 0QX, United Kingdom	0000-0003-3499-8262
Kenneth Haug	haug.kenneth@gmail.com	European Molecular Biology Laboratory, European Bioinformatics Institute (EMBL-EBI), Wellcome Genome Campus, Hinxton,	0000-0003-3168-4145

		Cambridge CB10 1SD, United Kingdom<br><br>Genome Research Limited,Wellcome Sanger Institute, Wellcome Trust Genome Campus, Hinxton, Saffron Walden CB10 1RQ, United Kingdom	
Massimiliano Izzo	massimiliano.izzo@oerc.ox.ac.uk	Oxford e-Research Centre, Department of Engineering Science, University of Oxford, 7 Keble Road, OX1 3QG, Oxford, United Kingdom	0000-0002-8100-6142
Martin Larralde	martin.larralde@embl.de	Structural and Computational Biology Unit, European Molecular Biology Laboratory (EMBL), Meyerhofstraße 1 69117 Heidelberg, Germany	0000-0002-3947-4444
Thomas N.<br><br>Lawson	thomas.nigel.lawson@gmail.com	School of Biosciences, University of Birmingham, Edgbaston, Birmingham, B15 2TT, United Kingdom	0000-0002-5915-7980
Alice Minotto	alice.minotto@earlham.ac.uk	Earlham Institute, Norwich Research Park, Norwich NR4 7UZ, United Kingdom	0000-0002-1670-1675
Pablo Moreno	pmoreno@ebi.ac.uk	European Molecular Biology Laboratory, European Bioinformatics Institute (EMBL-EBI), Wellcome Genome Campus, Hinxton, Cambridge CB10 1SD, United Kingdom	0000-0002-9856-1679
Venkata<br><br>Chandrasekhar<br><br>Nainala	venkata@ebi.ac.uk	European Molecular Biology Laboratory, European Bioinformatics Institute (EMBL-EBI), Wellcome Genome Campus, Hinxton, Cambridge CB10 1SD, United Kingdom	0000-0002-2564-3243
Claire O’Donovan	odonovan@ebi.ac.uk	European Molecular Biology Laboratory, European Bioinformatics Institute (EMBL-EBI), Wellcome Genome Campus, Hinxton, Cambridge CB10 1SD, United Kingdom	0000-0001-8051-7429
Luca Pireddu	luca.pireddu@crs4.it	Distributed Computing Group, CRS4: Center for Advanced Studies, Research & Development in Sardinia, Pula, Italy	0000-0002-4663-5613
Pierrick Roger	pierrick.roger@cea.fr	CEA, LIST, Laboratory for Data Analysis and Systems’ Intelligence, MetaboHUB, Gif-Sur-Yvette F-91191, France	0000-0001-8177-4873
Felix Shaw	felix.shaw@earlham.ac.uk	Earlham Institute, Norwich Research Park, Norwich NR4 7UZ, United Kingdom	0000-0001-9649-5906

Christoph Steinbeck	christoph.steinbeck@uni-jena.de	Cheminformatics and Computational Metabolomics, Institute for Analytical Chemistry, Lessingstr. 8, 07743 Jena, Germany	0000-0001-6966-0814
Ralf J. M. Weber	r.j.weber@bham.ac.uk	School of Biosciences, University of Birmingham, Edgbaston, Birmingham, B15 2TT, United Kingdom<br><br>Phenome Centre Birmingham, University of Birmingham, Edgbaston, Birmingham, B15 2TT, United Kingdom	0000-0002-8796-4771
Susanna-Assunta Sansone	susanna-assunta.sansone@oerc.ox.ac.uk	Oxford e-Research Centre, Department of Engineering Science, University of Oxford, 7 Keble Road, OX1 3QG, Oxford, United Kingdom	0000-0001-5306-5690
Philippe Rocca-Serra	philippe.rocca-serra@oerc.ox.ac.uk	Oxford e-Research Centre, Department of Engineering Science, University of Oxford, 7 Keble Road, OX1 3QG, Oxford, United Kingdom	0000-0001-9853-5668

Figures

Figure 1:

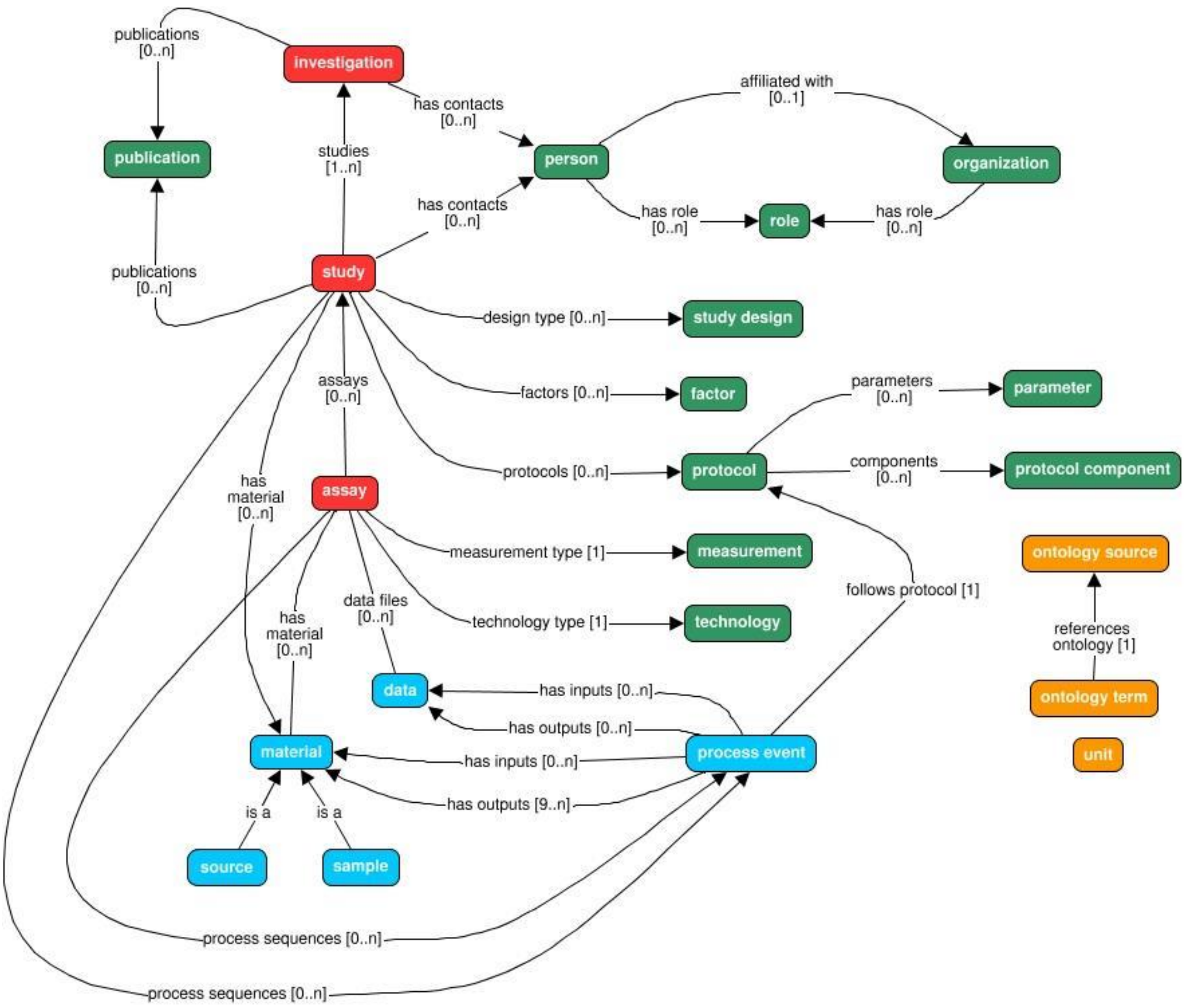

publications [0..n]
investigation
has contacts [0..n]
affiliated with [0..1]
publication
studies [1..n]
person
organization
has contacts [0..n]
has role [0..n]
role
has role [0..n]
publications [0..n]
study
design type [0..n]
study design
assays [0..n]
factors [0..n]
factor
parameters [0..n]
parameter
has material [0..n]
assay
protocols [0..n]
protocol
components [0..n]
protocol component
measurement type [1]
measurement
ontology source
follows protocol [1]
has material [0..n]
data files [0..n]
technology type [1]
technology
references ontology [1]
data
has inputs [0..n]
ontology term
has outputs [0..n]
material
has inputs [0..n]
process event
unit
has outputs [9..n]
is a
is a
source
sample
process sequences [0..n]
process sequences [0..n]

Figure 2:

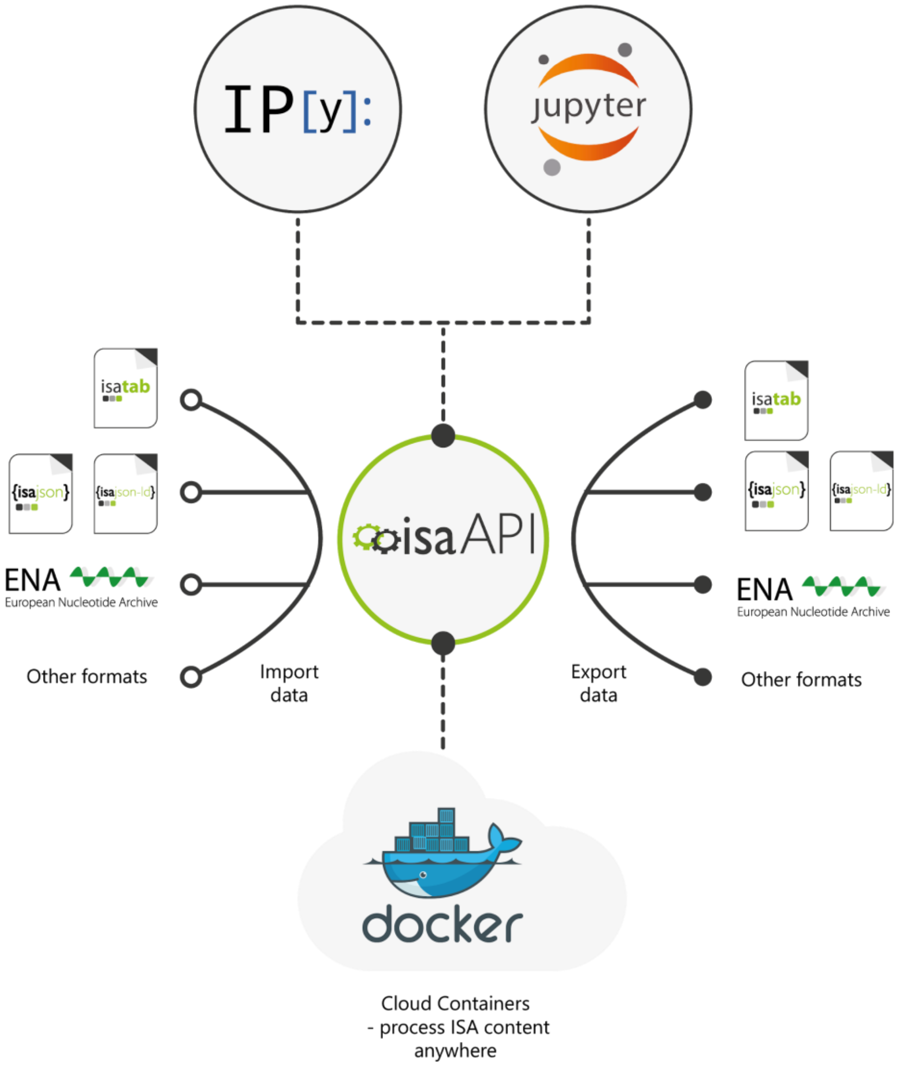
IP[y]:
jupyter
isatab
{isajson}
{isajson-ld}
ENA
European Nucleotide Archive
Other formats
Import
data
isaAPI
Export
data
isatab
{isajson}
{isajson-ld}
ENA
European Nucleotide Archive
Other formats
docker
Cloud Containers
- process ISA content
anywhere

Figure 3:

```python
#!/usr/bin/env python3

from isatools.model import *

def create_descriptor():
   """Returns a simple ISA-Tab 1.0 descriptor for demonstration."""
   investigation = Investigation()
   investigation.identifier = "i1"
   investigation.title = "My Simple ISA Investigation"

   study = Study(filename="s_study.txt")
   study.identifier = "s1"
   study.title = "My ISA Study"

   source = Source(name='source_material')
   study.sources.append(source)

   ncbitaxon = OntologySource(name='NCBITaxon', description="NCBI Taxonomy")
   characteristic_organism = Characteristic(category=OntologyAnnotation(term="Organism"),
                                 value=OntologyAnnotation(term="Homo Sapiens", term_source=ncbitaxon,
                                 term_accession="http://purl.bioontology.org/ontology/NCBITAXON/9606"))

   prototype_sample = Sample(name='sample_material', derives_from=[source])
   prototype_sample.characteristics.append(characteristic_organism)
   study.samples = batch_create_materials(prototype_sample, n=3)  # creates a batch of 3 samples

   sample_collection_protocol = Protocol(name="sample collection",
                                    protocol_type=OntologyAnnotation(term="sample collection"))
   study.protocols.append(sample_collection_protocol)

   sample_collection_process = Process(executes_protocol=sample_collection_protocol)
   sample_collection_process.inputs = study.sources
   sample_collection_process.outputs = study.samples

   study.process_sequence.append(sample_collection_process)

   investigation.studies.append(study)

   # Serialize the model that we built above to an ISA-Tab sent to stdout
   from isatools import isatab
   return isatab.dumps(investigation)

if __name__ == '__main__':
   print(create_descriptor())
```

Figure 4:

In [13]:
```
# make sure the extract, data file, and the processes are attached to the assay
assay.data_files.append(datafile)
assay.samples.append(sample)
assay.other_material.append(material)
assay.process_sequence.append(extraction_process)
assay.process_sequence.append(sequencing_process)
```

Finally, we attach the assay to the study.

In [14]:
```
study.assays.append(assay)
```

To write out the ISA-Tab, you can use the `isatab.dumps()` function:

In [15]:
```
from isatools import isatab
print(isatab.dumps(investigation))
```

```
Study Person Affiliation        University of Life
Study Person Roles      submitter
Study Person Roles Term Accession Number
Study Person Roles Term Source REF
--------
/var/folders/pv/4573yvqx31x_2dwbj9g0cn8c0000gn/T/tmp4ap0tj8o/s_study.txt
Source Name     Protocol REF    Sample Name     Characteristics[Organism]       Term Source REF Term Accession Number
source_material sample collection       sample_material-2       Homo Sapiens    NCBITaxon       http://purl.bioontolo
gy.org/ontology/NCBITAXON/9606
source_material sample collection       sample_material-1       Homo Sapiens    NCBITaxon       http://purl.bioontolo
gy.org/ontology/NCBITAXON/9606
source_material sample collection       sample_material-0       Homo Sapiens    NCBITaxon       http://purl.bioontolo
gy.org/ontology/NCBITAXON/9606
--------
/var/folders/pv/4573yvqx31x_2dwbj9g0cn8c0000gn/T/tmp4ap0tj8o/a_assay.txt
Sample Name     Protocol REF    Extract Name    Protocol REF    Raw Data File
sample_material-0       extraction      extract-0       sequencing      sequenced-data-0
sample_material-1       extraction      extract-1       sequencing      sequenced-data-1
sample_material-2       extraction      extract-2       sequencing      sequenced-data-2
```

Figure 5:

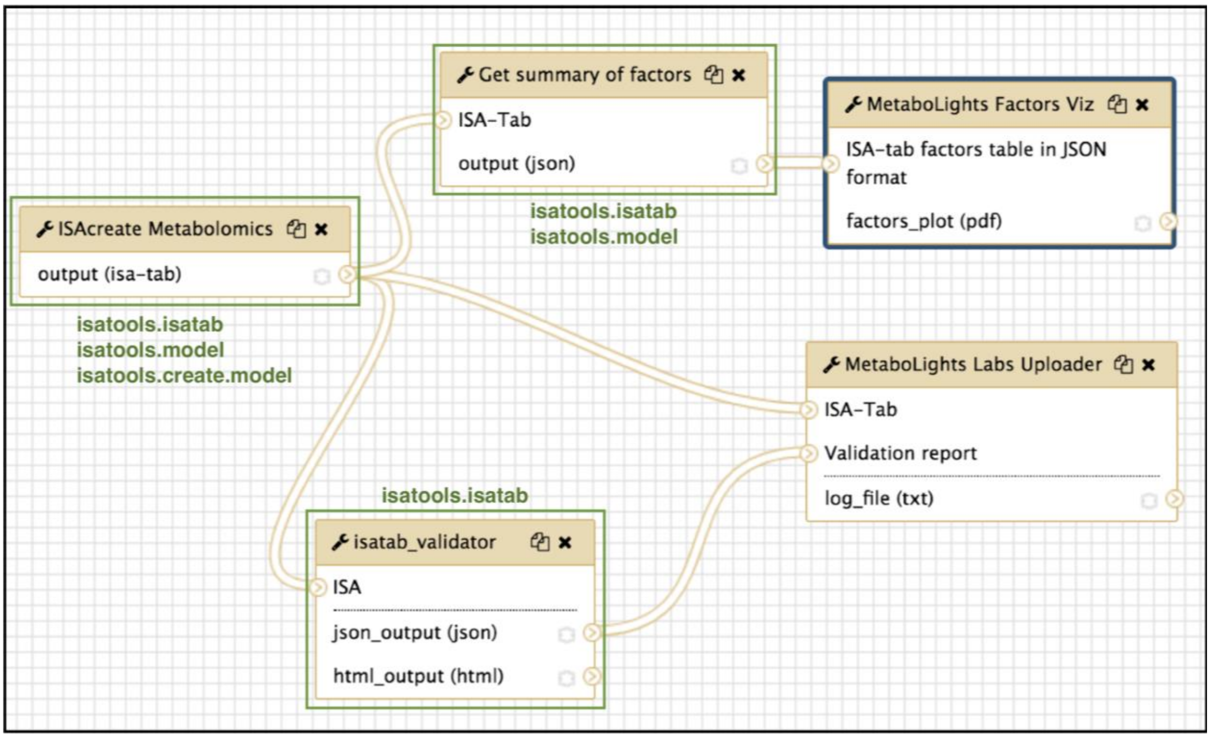

Figure 6:

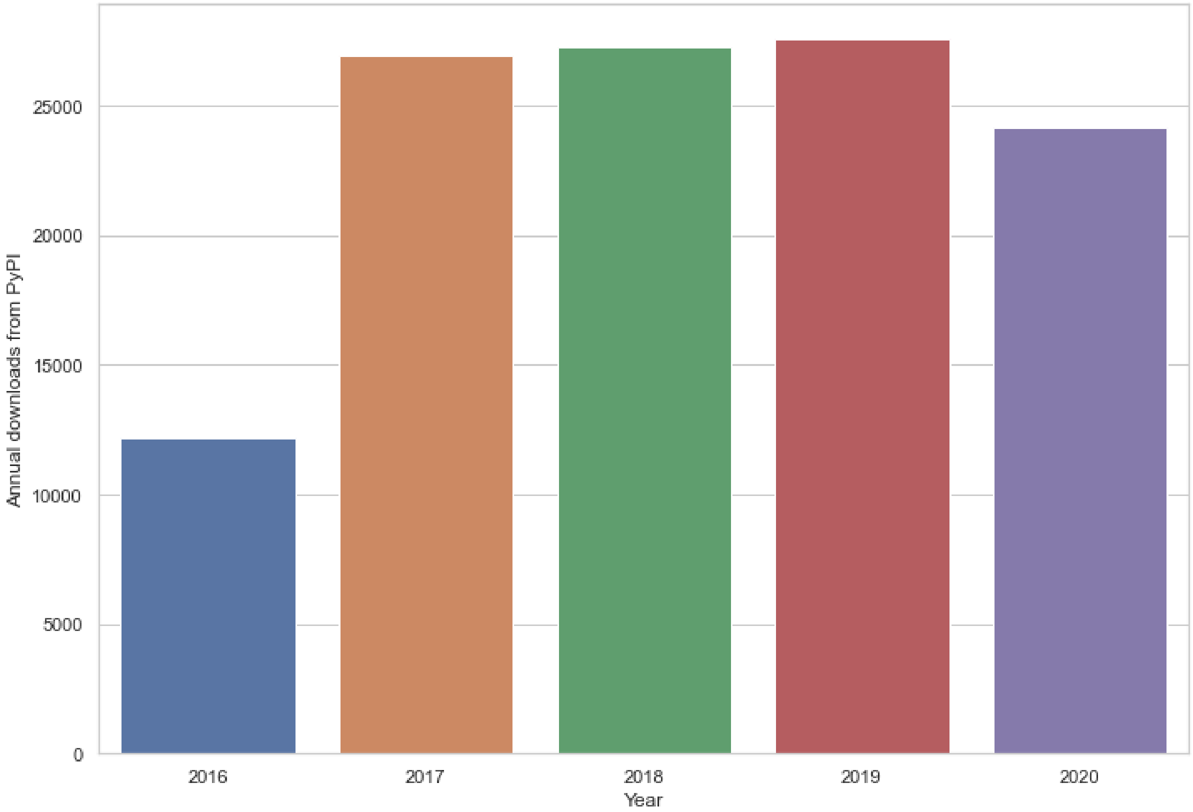

Annual downloads from PyPI
0
5000
10000
15000
20000
25000
2016
2017
2018
2019
2020
Year

Figure 7:

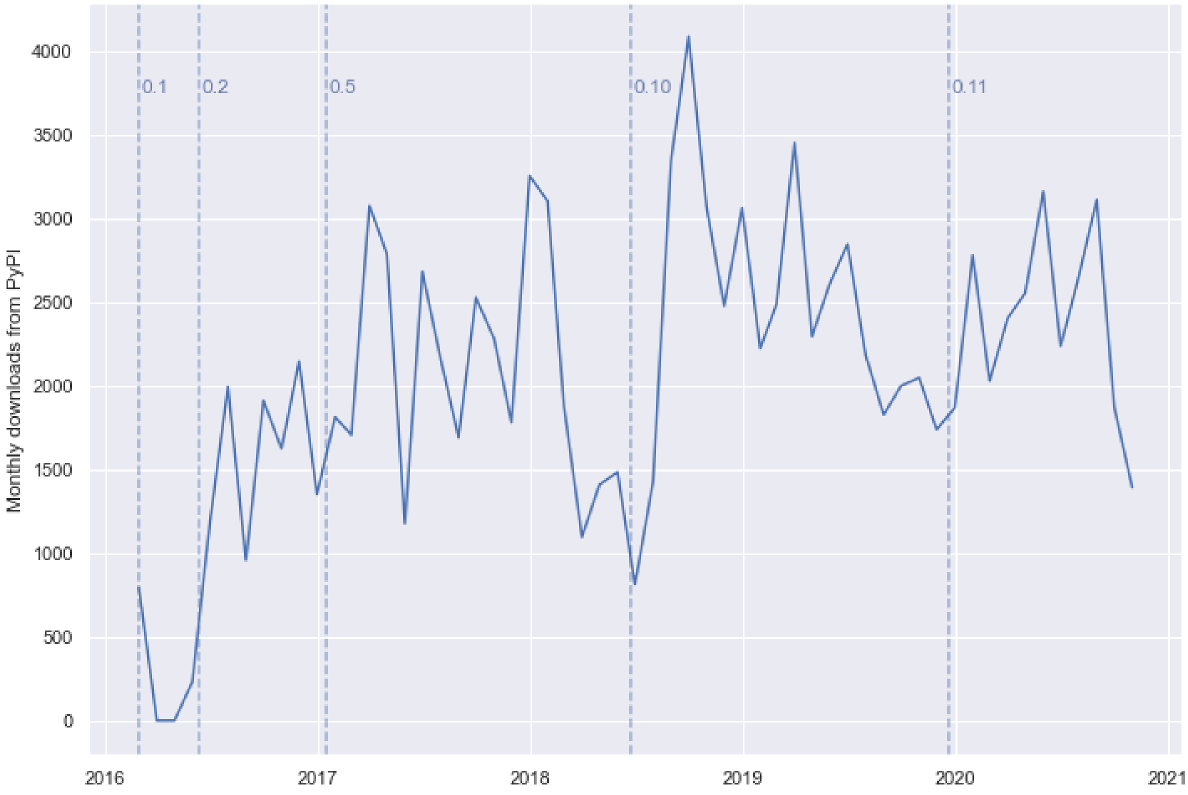

Monthly downloads from PyPI
0
500
1000
1500
2000
2500
3000
3500
4000
0.1
0.2
0.5
0.10
0.11
2016
2017
2018
2019
2020
2021

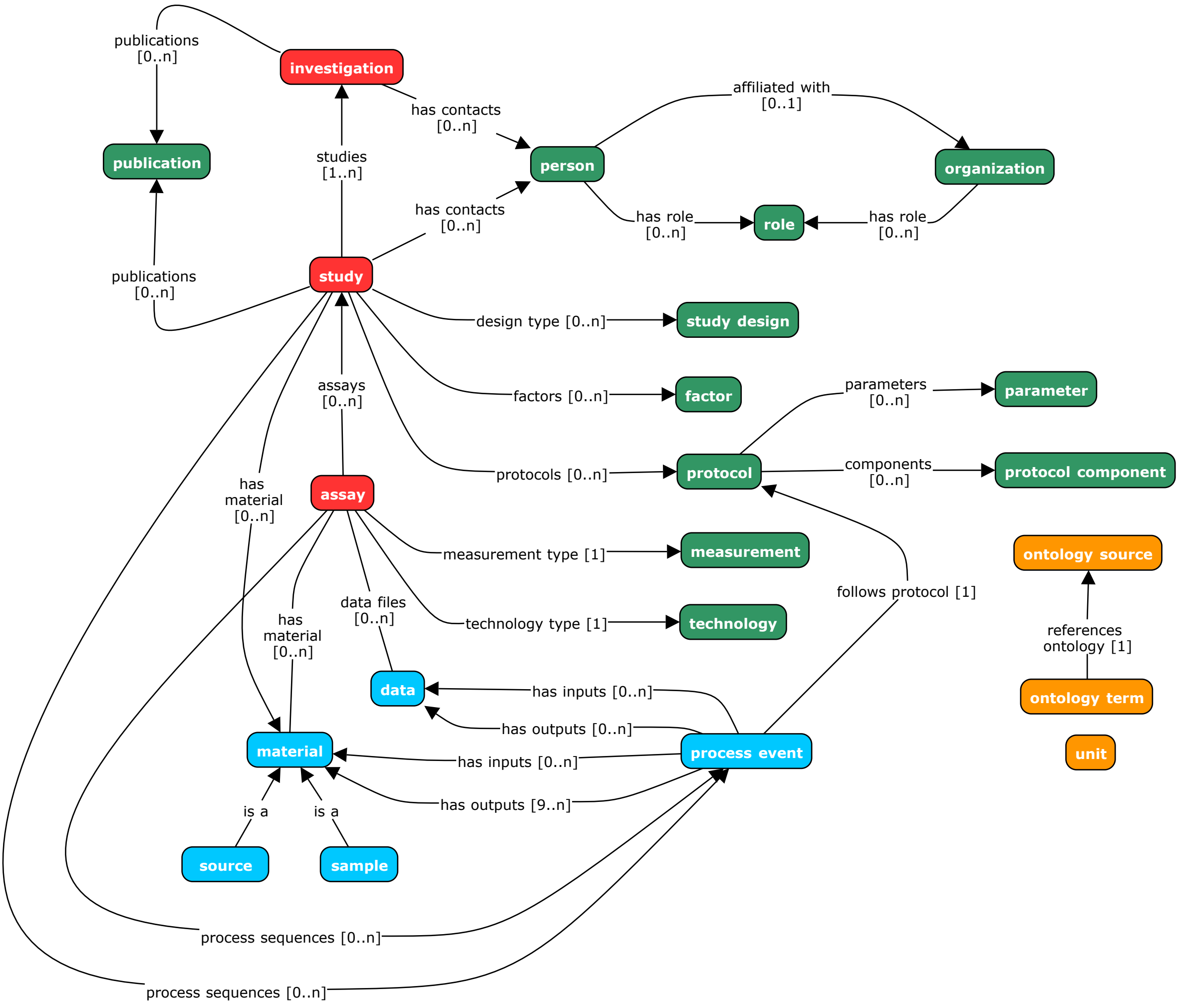
investigation
publications [0..n]
publication
studies [1..n]
has contacts [0..n]
person
affiliated with [0..1]
organization
has role [0..n]
role
has role [0..n]
has contacts [0..n]
study
publications [0..n]
design type [0..n]
study design
factors [0..n]
factor
assays [0..n]
parameters [0..n]
parameter
protocols [0..n]
protocol
components [0..n]
protocol component
has material [0..n]
assay
measurement type [1]
measurement
ontology source
follows protocol [1]
data files [0..n]
has material [0..n]
technology type [1]
technology
references ontology [1]
data
has inputs [0..n]
ontology term
has outputs [0..n]
material
has inputs [0..n]
process event
unit
has outputs [9..n]
is a
is a
source
sample
process sequences [0..n]
process sequences [0..n]

Figure 2

Click here to access/download;Figure;ISA API paper - Figure 2.png

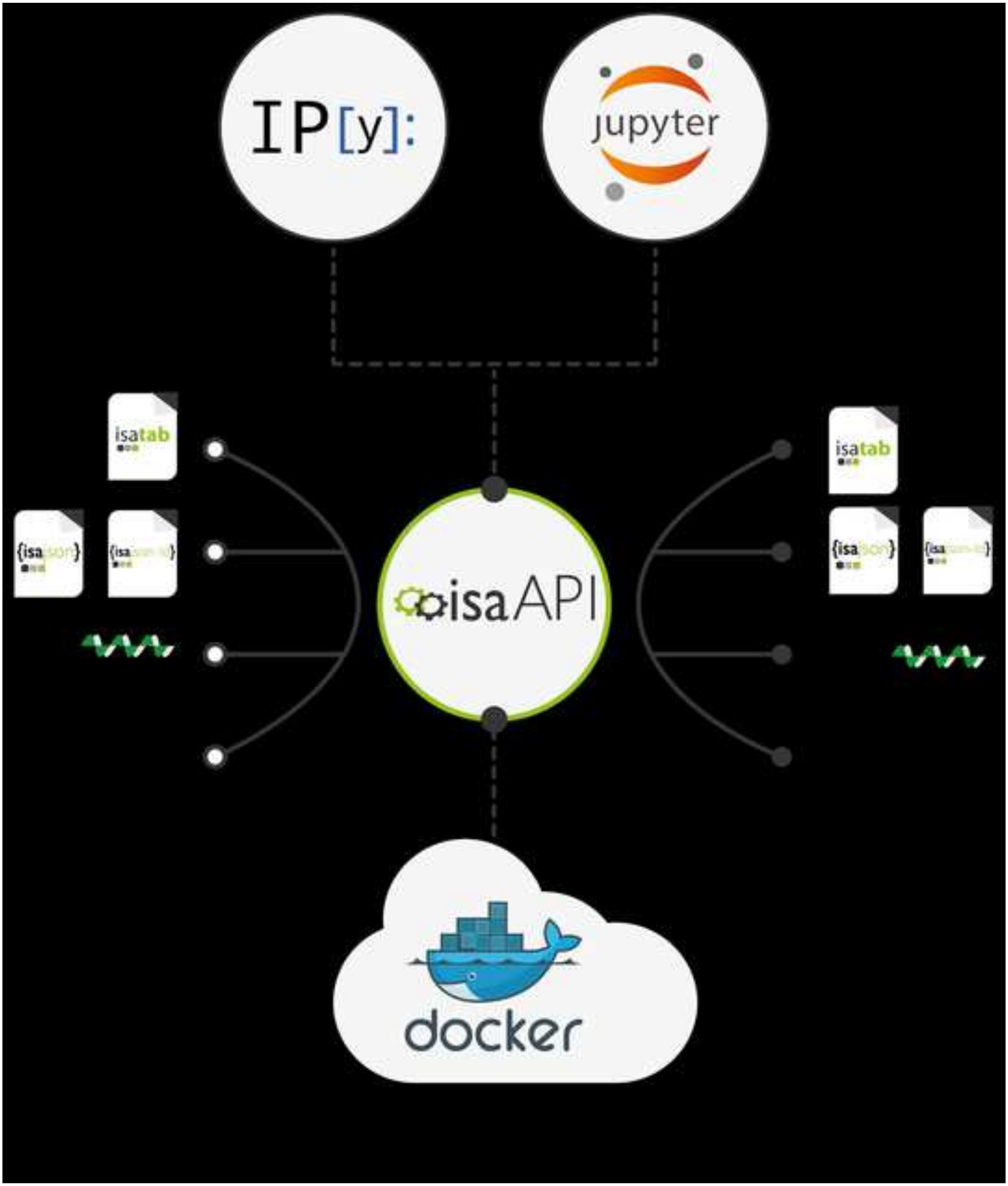

 Click here to access/download;Figure;ISA API paper - Figure 3.png

In [13]:
```
# Ensure Processes are linked forward and backward. plink(from_process, to_process) is a function to set
# these links for you. It is found in the isatools.model package
plink(extraction_process, sequencing_process)

# make sure the extract, data file, and the processes are attached to the assay
assay.data_files.append(datafile)
assay.samples.append(sample)
assay.other_material.append(material)
assay.process_sequence.append(extraction_process)
assay.process_sequence.append(sequencing_process)
```

Finally, we attach the assay to the study.

In [14]:
```
study.assays.append(assay)
```

To write out the ISA-Tab, you can use the `isatab.dumps()` function:

In [15]:
```
from isatools import isatab
print(isatab.dumps(investigation))
```

```
Study Person Affiliation        University of Life
Study Person Roles      submitter
Study Person Roles Term Accession Number
Study Person Roles Term Source REF
--------
/var/folders/pv/4573yvqx31x_2dwbj9g0cn8c0000gn/T/tmpaa2z7thm/s_study.txt
Source Name     Protocol REF     Sample Name     Characteristics[Organism]      Term Source REF Term Accession Number
source_material sample collection       sample_material-2       Homo Sapiens    NCBITaxon       http://purl.bioontolo
gy.org/ontology/NCBITAXON/9606
source_material sample collection       sample_material-0       Homo Sapiens    NCBITaxon       http://purl.bioontolo
gy.org/ontology/NCBITAXON/9606
source_material sample collection       sample_material-1       Homo Sapiens    NCBITaxon       http://purl.bioontolo
gy.org/ontology/NCBITAXON/9606
--------
/var/folders/pv/4573yvqx31x_2dwbj9g0cn8c0000gn/T/tmpaa2z7thm/a_assay.txt
Sample Name     Protocol REF     Extract Name     Protocol REF     Raw Data File
sample_material-0       extraction      extract-0       sequencing      sequenced-data-0
sample_material-1       extraction      extract-1       sequencing      sequenced-data-1
sample_material-2       extraction      extract-2       sequencing      sequenced-data-2
```

```python
#!/usr/bin/env python3

from isatools.model import *

def create_descriptor():
    """Returns a simple ISA-Tab 1.0 descriptor for demonstration."""
    investigation = Investigation()
    investigation.identifier = "i1"
    investigation.title = "My Simple ISA Investigation"

    study = Study(filename="s_study.txt")
    study.identifier = "s1"
    study.title = "My ISA Study"

    source = Source(name='source_material')
    study.sources.append(source)

    ncbitaxon = OntologySource(name='NCBITaxon', description="NCBI Taxonomy")
    characteristic_organism = Characteristic(category=OntologyAnnotation(term="Organism"),
                                 value=OntologyAnnotation(term="Homo Sapiens", term_source=ncbitaxon,
                                 term_accession="http://purl.bioontology.org/ontology/NCBITAXON/9606"))

    prototype_sample = Sample(name='sample_material', derives_from=[source])
    prototype_sample.characteristics.append(characteristic_organism)
    study.samples = batch_create_materials(prototype_sample, n=3)  # creates a batch of 3 samples

    sample_collection_protocol = Protocol(name="sample collection",
                                          protocol_type=OntologyAnnotation(term="sample collection"))
    study.protocols.append(sample_collection_protocol)

    sample_collection_process = Process(executes_protocol=sample_collection_protocol)
    sample_collection_process.inputs = study.sources
    sample_collection_process.outputs = study.samples

    study.process_sequence.append(sample_collection_process)

    investigation.studies.append(study)

    # Serialize the model that we built above to an ISA-Tab sent to stdout
    from isatools import isatab
    return isatab.dumps(investigation)

if __name__ == '__main__':
    print(create_descriptor())
```

Figure 5

Click here to access/download;Figure;ISA API paper - Figure 5.png

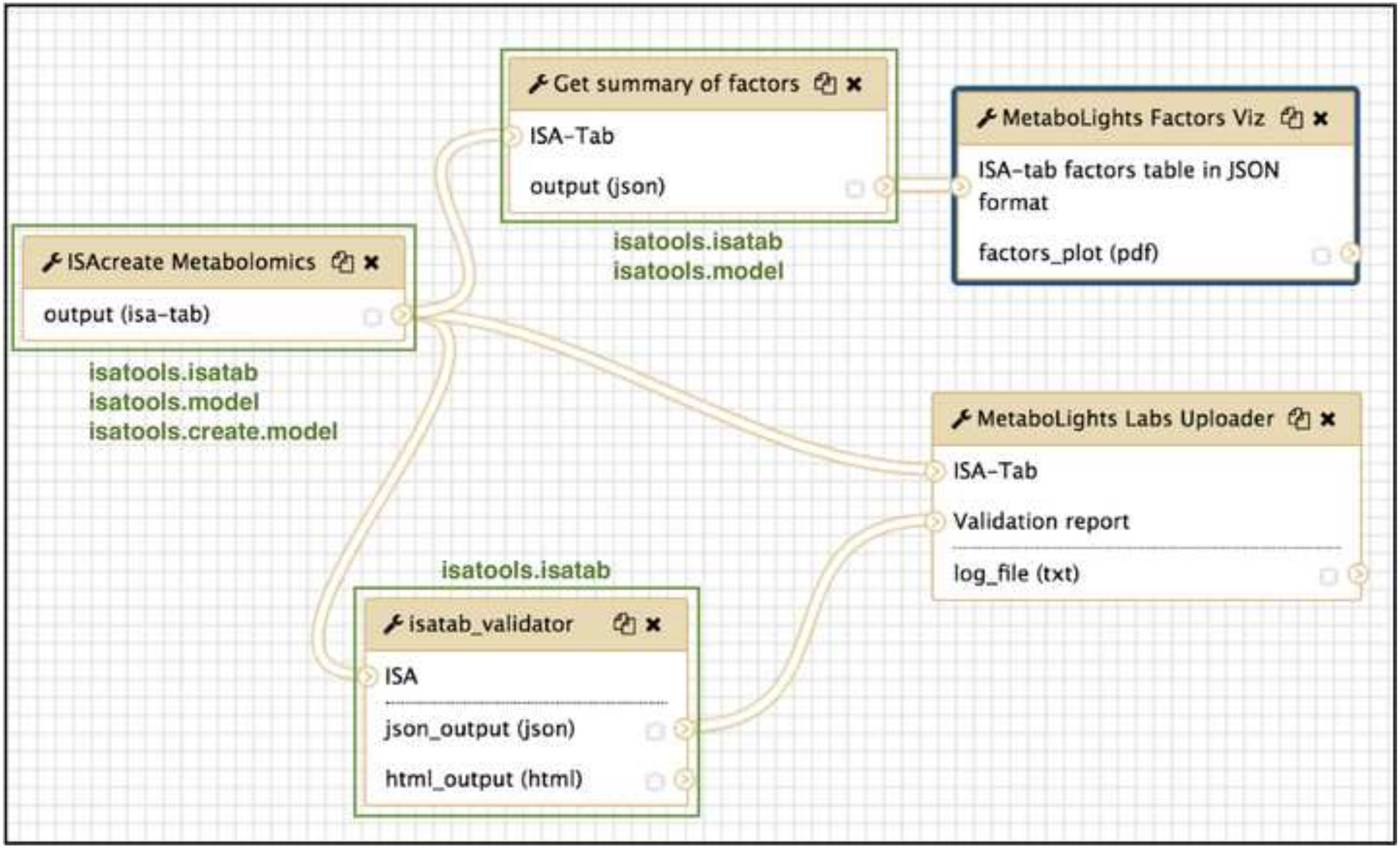

Figure 6

Click here to access/download;Figure;fig6.png

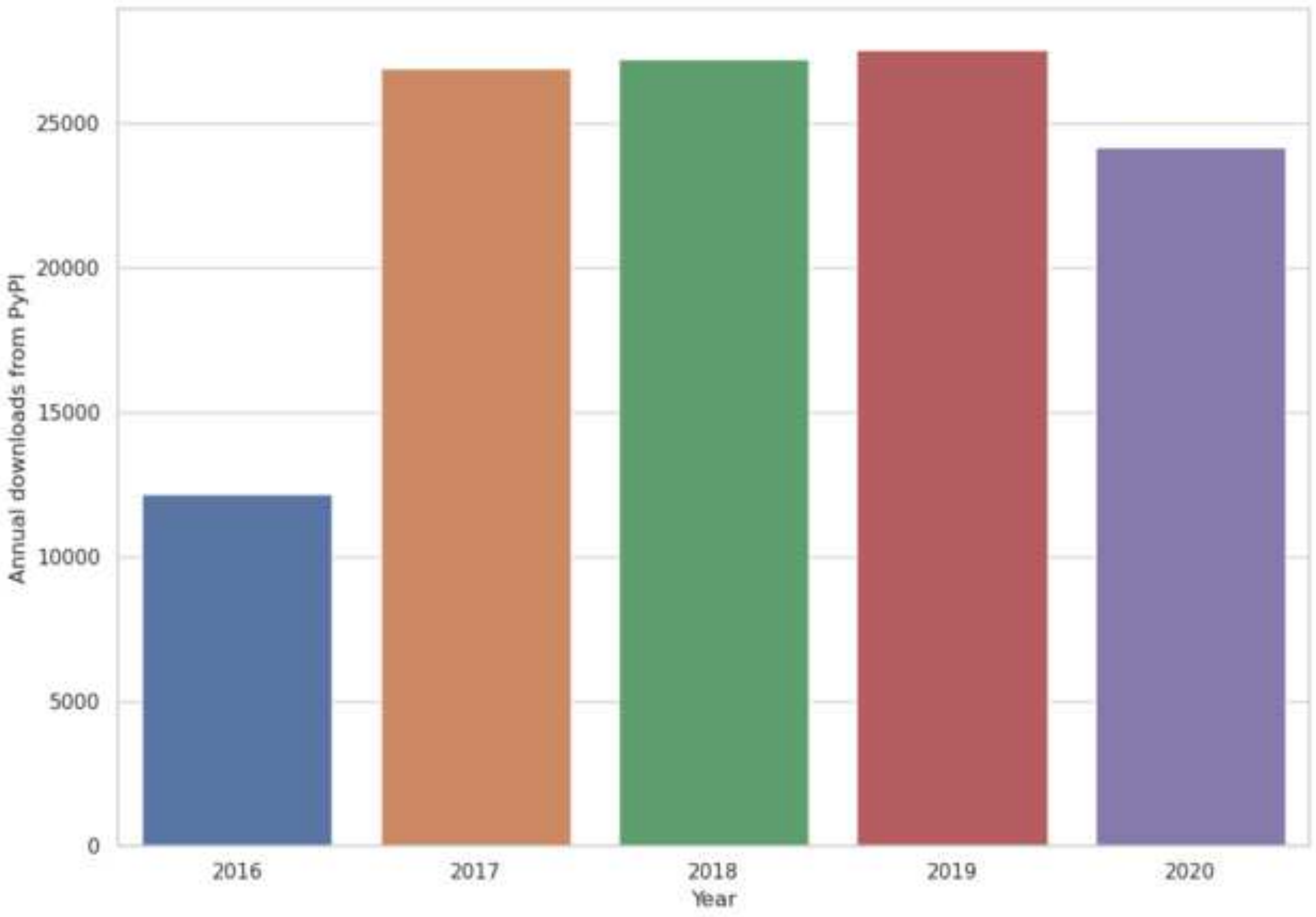

Figure 7

Click here to access/download;Figure;fig7.png

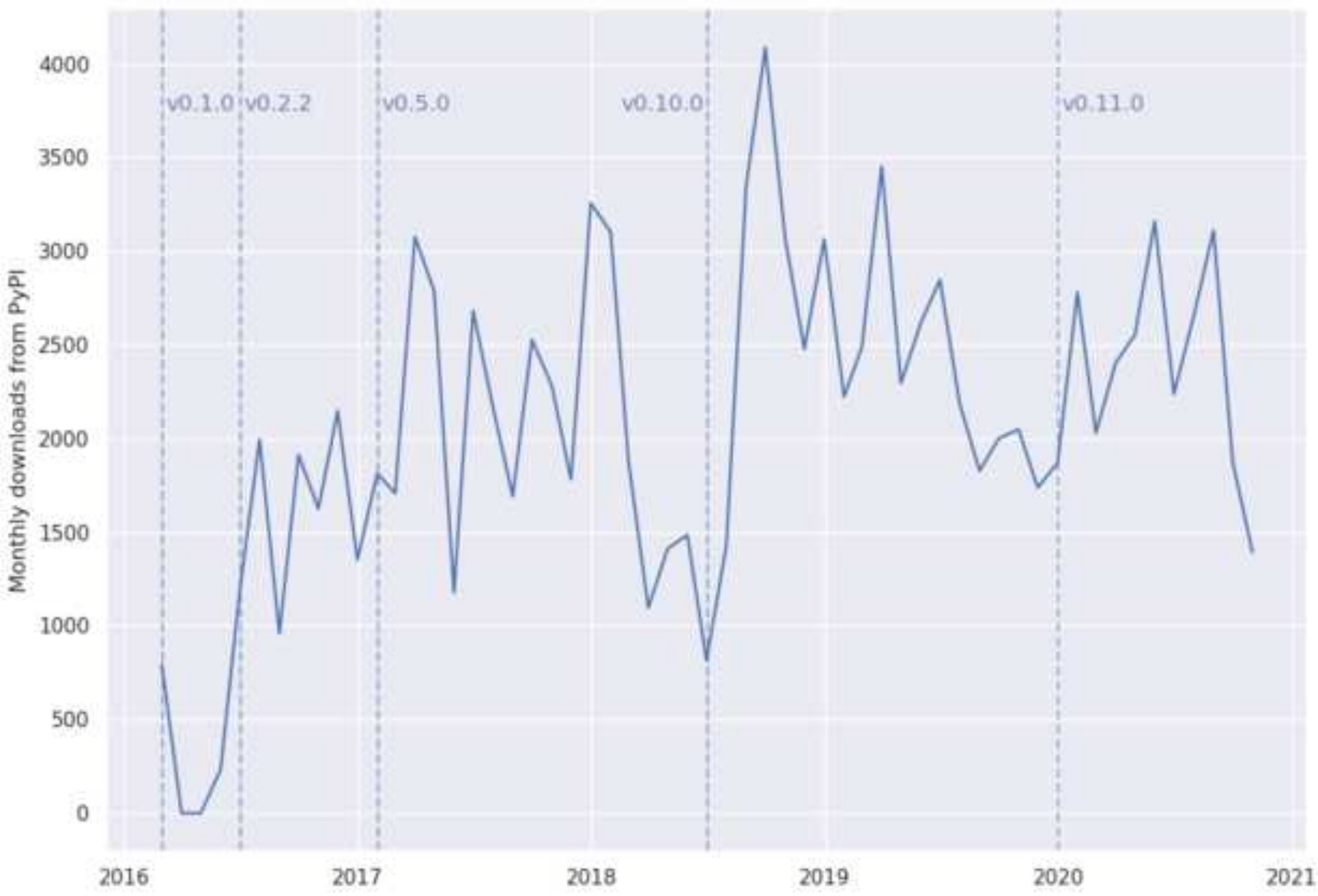

Supplement: giab060_GIGA-D-20-00333_Revision_1 [file giab060_giga-d-20-00333_revision_1.pdf]
