# Supplementary material for: ISA API: An open platform for interoperable life science experimental metadata
Source: Gigascience. 2021 Sep 16;10(9):giab060. doi: 10.1093/gigascience/giab060 (PMC8444265; doi:10.1093/gigascience/giab060)
Supplement: giab060_Response_to_Reviewer_Comments_Original_Submission [file giab060_response_to_reviewer_comments_original_submission.pdf]

# Author's Response To Reviewer Comments

Close

Editor comment: Your manuscript "ISA API: An open platform for interoperable life science experimental metadata" (GIGA-D-20-00333) has been assessed by our reviewers. Although it is of interest, we are unable to consider it for publication without some additional work. The reviewers have raised a number of points which we believe would improve the manuscript and would allow a revised version to be published in GigaScience.

Author response: First, we thank the reviewers for their time and expertise to appraise our work and we are grateful for the opportunity given to improve our manuscript, software and to clarify any misunderstandings. We enclose henceforth our responses to each of the comments, as well as indications of the changes, where we agree a change is warranted, to the initial manuscript to address specific comments.

Editor comment: In addition, please register any new software application in the bio.tools and SciCrunch.org databases to receive RRID (Research Resource Identification Initiative ID) and biotoolsID identifiers, and include these in your manuscript. This will facilitate tracking, reproducibility and re-use of your tool.

Author response: We have added the biotoolsID and SciCrunch RRID to the "Availability of source code and requirements" section found on page 20.

Editor comment: Please include a point-by-point within the 'Response to Reviewers' box in the submission system. Please ensure you describe additional experiments that were carried out and include a detailed rebuttal of any criticisms or requested revisions that you disagreed with. Please also ensure that your revised manuscript conforms to the journal style, which can be found in the Instructions for Authors on the journal homepage. If the data and code has been modified in the revision process please be sure to update the public versions of this too.

Author response: In our 'Response to Reviewers' we describe our rebuttals of any points we disagreed with and also point to the pages where we made changes in the draft manuscript for the points we do agree with. The revised manuscript remains styled as per the Instructions to Authors. No data and code has been modified in the revision process.

Responses to Reviewer 1
Reviewer 1 remarks: In the paper "ISA API: An open platform for interoperable life science experimental metadata" Johnson et al. present a extensive Python API for reading, writing and handling of metadata in the ISA format. The authors describe the increasing use of the ISA formats and thus indicate the need for better tools to handle such data. The article is well written and good to understand.

The ISA tools package contains extensive functionality and a solid documentation. Furthermore, it can be installed with PyPi and Bioconda, which I think should be standard nowadays. The authors furthermore provide a docker image, which is nice. All in all, I think the ISA tools package is a genuinely useful piece of software that is well written, which is why I recommend this manuscript for publication in GigaScience.

Author response: We appreciate Reviewer 1's comments and are heartened that they felt the article is well written and recommended for publication in GigaScience. Below we address their comments point-by-point:

Reviewer 1 comment: I would like to know whether support for the upload to additional databases will be added in the future - this could be noted in the text.

Author response: As a free and open source software library, an objective of ISA API is to develop any new features the ISA user community proposes. Beyond the current export functionality, we fully expect to continue supporting data deposition to EMBL-EBI data repositories and also are working in close collaboration with FAIRdom/SEEK.

Author changes: We prefer not to speculate on future work too specifically in the manuscript, but we have added a line to state "Support for submission to additional databases will be developed in future" on page 8.

Reviewer 1 comment: The article contains many figures with only little content. I would strongly advise to merge some of these figures into a smaller subset of figures to improve the readability.

Author response: We have reviewed the manuscript's figures. Figures 1-5 contain text and we believe those figures would not be very readable if reduced. We think this can be addressed further if necessary in the typesetting phase of manuscript production, as the draft manuscript is presented without any typeset formatting as per the GigaScience manuscript preparation guidelines in https://academic.oup.com/gigascience/pages/instructions_to_authors#Preparing%20Main%20Manuscript%20Text.

Author changes: We have merged figures 6 and 7 (download stats) into a single figure as two side-by-side panels found on page 15.

Reviewer 1 comment: The authors spend a considerable amount of text on download statistics - something that in my opinion is not really that relevant for the software package. I would recommend to considerably shorten this section.

Author changes: We have now considerably shortened this section on pages 15 and 16.

Reviewer 1 comment: On a similar note, the methods section basically just describes how these download statistics were handled. Considering this article describes a software package, it might be more useful to the reader (and reviewer) to elaborate a bit on how the software is written, maintained, structured, tested - and related things.

Author changes: We have added a discussion about our development approaches in the "Methods" section of the manuscript on pages 17-19 and also about ISA API's documentation on page 13.

Responses to Reviewer 2
Reviewer 2 remarks: The authors describe the Python library "isatools" for accessing ISA (investigation study assay) files in ISA-tab and ISA-json format. The authors start by sdescribing their previous work around the ISA data model and file formats in detail. They then describe their implementation and the features of their API. They highlight the extensibility and efficiency of their object oriented model. They describe in detail how meta data can be curated in ontologies and that currently extensions are underway for the assisted creation of study meta data. They then refer to early adopters and a stable and growing community. They conclude with the statement that their library is "a major step forward in making the ISA framework open and interoperable".

Overall, we have found the ISA data model and ISA-tab data format to be very useful in our own work. However, there are some issues with the software including apparent bugs as described below. In 2018, my colleagues and me considered using ISA-API in our project for ISA-Tab parsing but the problems and the lack of automated tests made us roll our own (also see below).

Overall, the authors make a clear point, the paper is well-written. However, the software appears to be unfinished and some work is required to make it suitable for publication.

Author response: We appreciate Reviewer 2's comments on the manuscript and for sharing their opinions about the software based on their personal experiences, and are happy to hear that they state that the paper is well-written. We disagree that the software is unfinished and rebut the reviewer's specific points on that issue in our responses below.

Below we address their specific comments point-by-point:

Reviewer 2 comment: The ISA-creator and Bio-GraphIIn are cited as "helped grow the ISA community of users". The authors should offer evidence for this as

(a) by our own experience ISA-creator is very hard to use and this is also reflected by the expressed opinion on ISA-creator by anyone I have met so far who has used it and

(b) it is not possible to validate how Bio-GraphII has helped grow the community as the website linked to in the cited article is not available anymore and no source code is available, e.g., on Github. The Google groups forum has less than 10 threads per year, with 2 in 2020 so far and one in 2019. The authors should balance these counts with their "PyPi" download counts statistics.

Author response: ISAcreator has been the de facto reference implementation of the ISA-Tab specification. From our own experience, we know that researchers have been consistently using ISAcreator over many years to

prepare their data submissions for various databases. This includes Metabolights (EMBL-EBI), GenomeSpace (Broad Institute), Toxbank (EU), and Genelab (NASA) to name just a few.

We agree that Bio-GraphIIn should in fact not be mentioned as it has been long retired. This should instead have referenced the BioInvestigation Index (BII) database software, which has been used by various organisations worldwide including the UK NERC Environmental Bioinformatics Centre, the US Personalized NSAID Therapeutics Consortium (PENTACON), and the Harvard Stem Cell Discovery Engine. There is evidence of BII still in use - the aforementioned organisations continue to maintain running instances.

The known ISA community is listed in the ISA Commons, which is referenced in the manuscript.

We disagree that Google groups activity can be indicative of ISA community activity. The ISA Google group has historically had low activity owing to it being a secondary point of contact to the direct email address to the ISA team.

Author changes: We have replaced reference to Bio-GraphIIn with BioInvestigation Index on page 5.

Reviewer 2 comment: The authors should cite other published APIs for ISA file formats, e.g., AltamISA.

- Kuhring, Mathias, et al. "AltamISA: a Python API for ISA-Tab files." Journal of Open Source Software 4.40 (2019): 1610.

Author response: We agree that references to other published ISA-related software should be included and appreciate that the reviewer suggests that we cite his own work.

Author changes: We additionally believe that we should make reference to precursor Perl and Python projects that we are also aware of. We have added a new "Related work" subsection at the end of the Background section beginning on page 6 that references Bio-Parser-ISATab, biopy-isatab, AltamISA and isa4j.

Reviewer 2 comment: The authors should show proof for "efficiency" of their object-oriented model, e.g., by comparing import efficiency with that of altamISA. I'm raising this point as some users raised questions on efficiency when loading/writing data files in the ISA-API Github Issues.

Author response: The use of the word "efficient" in the manuscript is about the representation of ISA content in the data model and not about the processing performance (ie. when doing i/o). We do not intend to present in this paper any performance evaluation/comparison of ISA API with other software that is not comparable in terms of features. As far as we understand, AltamISA and isa4j focus only on ISA-Tab. ISA API has a much broader scope to interoperate between ISA formats and other formats.

Author changes: We have updated the single use of the word "efficient" to "coherent" in the relevant subsection heading on page 9 to avoid confusion to the reader. Efficiency is not discussed in the manuscript.

Reviewer 2 comment: The authors write that development is in progress but it appears from the Github code frequency graph that development has mostly stalled since 2018.

Author response: We disagree that development has stalled since 2018 and disagree that the GitHub code frequency graph is a good measure of development.

The GitHub code frequency graph is unrepresentative of activity as it is weighted on the numbers of lines of code (LOC) added/removed. LOC may indicate the degree of changes to source code but we believe that even small changes indicate that a software is being actively maintained or updated. A better view of activity may be the graph of contributions to the master branch (https://github.com/ISA-tools/isa-api/graphs/contributors) that counts commits rather than LOC and clearly shows activity since 2018.

We also believe development work goes beyond activity shown by GitHub commits, such as testing, feature requests, and collaborating with the user community who are using/developing with ISA API as development, activity of which is not always reflected on GitHub.

We can confirm to the reviewer that development continues and has not stalled since 2018.

Reviewer 2 comment: The authors should explain in more detail how stable their API is and what the limitations and assumptions are. In my opinion, one important point in data import and export is looking how data looks after

a "round-trip", e.g., import ISA-Tab, followed by export ISA-Tab.

I have done this on the official ISA data sets (https://github.com/ISA-tools/ISAdatasets, commit f20be4f83dc5f6f7ec419bfd634efba3177e4ae4). Here are the (to me unexpected results for official example data):

(a) On BII-I-1, whole columns disappear such as the first "Material Type" column,
(b) All other datasets fail to parse and parsing crashes with Python exceptions.

I think the authors should work on these points. It cannot be judged whether the software can be published this point. The software appears unfinished and some more work has to go into it to allow for publication.

Author response: We disagree that the software appears unfinished.

While we appreciate that the reviewer has carried out their own round-tripping tests, we cannot respond to an informally conducted test with very few details revealed to us. We can point out to the reviewer that the ISA datasets commit they reference in their review is not on the test data branch of which ISA API uses in the project's automated test suite. Round-tripping only works as a test if you assume the input is valid to begin with. Additionally, round-tripping is a narrow case that does not represent the lion's share of use cases for ISA API, which is to interoperate ISA formats with other formats.

We encourage the reviewer to actively engage with the open source ISA API project to understand how ISA API's test infrastructure works, and so that they can report bugs, or contribute fixes if they have tried to fix any bugs. The project has been open source since its inception in 2015 and has always universally welcomed contributions/discussions with existing or potential users.

The point is therefore more an issue of documentation than implementation. Pretty much all projects can be faulted on the documentation. This is also to address some of these issues that the ISA API documentation has been migrated from readthedocs to the more modern jupyterbook infrastructure.

We strongly believe that based on our user community's adoption of the ISA API (the major adopters of which are described in the paper), and the sustained and growing number of downloads via PyPI, that the software is stable and being used in production by third parties.

Reviewer 2 comment: The authors should provide more automated tests for their software. In 2018 when we tried out the package we found some inconsistencies and problems but found it hard to fix bugs in the large body of software because of the lack of comprehensive automated tests.

Author response: We disagree that there is a lack of comprehensive automated tests and are sorry to hear that they found it hard to fix bugs in 2018.

Today, ISA API today has 631 automated unit tests on the master branch of the GitHub project. When bugs are reported, we usually add a test relating to the bug in our automated test suite to trace and fix bugs in a TDD process.

Where there are any issues found, we warmly welcome everybody to log any issues and make fix/feature requests, and to contribute/collaborate with fixes/features, via our open source GitHub project.

Author changes: We have added a discussion about our development approaches in the "Methods" section of the manuscript (also suggested by Reviewer 1) on pages 17-19.

Close
