# Supplementary material for: ISA API: An open platform for interoperable life science experimental metadata
Source: Gigascience. 2021 Sep 16;10(9):giab060. doi: 10.1093/gigascience/giab060 (PMC8444265; doi:10.1093/gigascience/giab060)
Supplement: giab060_Response_to_Reviewer_Comments_Revision_1 [file giab060_response_to_reviewer_comments_revision_1.pdf]

Author's Response To Reviewer Comments

Close

Dear Editor

We would like to thank you and all reviewers for their time, comments and insights. We have completed the revision to the manuscript, which mainly consists in updating figure 4 and its associated caption.
The main changes actually affect the ISA-API itself, with modifications significantly improving two areas:
• code-wise: speed of serialization to ISA-Tab, especially noticeable with large experiments, as reported by collaborators using the ISA-API in the field of Plant Science (Breeding Application Programming Interface BRAPI). The improvement to the algorithm was carried out by Dominique Batista, in collaboration with Massimiliano Izzo and myself. On this ground, Mr Batista has been included as author for his excellent contribution.
• documentation-wise: As suggested by Reviewer #3, we have now added a new jupyter notebook (https://isa-tools.org/isa-api/content/notebooks/programmatic-rebuildof-BII-S-3-with-ISA-API.html) documenting round trip example, as part of our online documentation known as the `the ISA-cookbook` (a jupyterbook). This new new notebook demonstrates the ability to create an ISA document, write it to ISA-Tab format, read it back to memory and serialize it back to ISA-tab, thus demonstrating the correctness of the parsing.

The ISA-API release 10.3, to be released this month, will be carrying those changes.

Once again, on behalf of my co-authors, I would like to thank all those involved in the process.

Kind regards

Dr Rocca-Serra

Close
