# Supplementary material for: ISA API: An open platform for interoperable life science experimental metadata
Source: Gigascience. 2021 Sep 16;10(9):giab060. doi: 10.1093/gigascience/giab060 (PMC8444265; doi:10.1093/gigascience/giab060)
Supplement: giab060_Reviewer_1_Report_Original_Submission — Kevin Menden -- 12/9/2020 Reviewed [file giab060_reviewer_1_report_original_submission.pdf]

Reviewer Report

Title: ISA API: An open platform for interoperable life science experimental metadata

Version: Original Submission **Date:** 12/9/2020

Reviewer name: Kevin Menden

Reviewer Comments to Author:

In the paper "ISA API: An open platform for interoperable life science experimental metadata" Johnson et al. present a extensive Python API for reading, writing and handling of metadata in the ISA format. The authors describe the increasing use of the ISA formats and thus indicate the need for better tools to handle such data. The article is well written and good to understand.

The ISA tools package contains extensive functionality and a solid documentation. Furthermore, it can be installed with PyPi and Bioconda, which I think should be standard nowadays. The authors furthermore provide a docker image, which is nice. All in all, I think the ISA tools package is a genuinely useful piece of software that is well written, which is why I recommend this manuscript for publication in GigaScience.

However, a few minor things should be changed. Personally, I would like to know whether support for the upload to additional databases will be added in the future - this could be noted in the text.

The article contains many figures with only little content. I would strongly advise to merge some of these figures into a smaller subset of figures to improve the readability.

The authors spend a considerable amount of text on download statistics - something that in my opinion is not really that relevant for the software package. I would recommend to considerably shorten this section.

On a similar note, the methods section basically just describes how these download statistics were handled. Considering this article describes a software package, it might be more useful to the reader (and reviewer) to elaborate a bit on how the software is written, maintained, structured, tested - and related things.

Level of Interest

Please indicate how interesting you found the manuscript: Choose an item.

Quality of Written English

Please indicate the quality of language in the manuscript: Choose an item.

Declaration of Competing Interests

Please complete a declaration of competing interests, considering the following questions:

- Have you in the past five years received reimbursements, fees, funding, or salary from an organisation that may in any way gain or lose financially from the publication of this manuscript, either now or in the future?
- Do you hold any stocks or shares in an organisation that may in any way gain or lose financially from the publication of this manuscript, either now or in the future?
- Do you hold or are you currently applying for any patents relating to the content of the manuscript?
- Have you received reimbursements, fees, funding, or salary from an organization that holds or has applied for patents relating to the content of the manuscript?
- Do you have any other financial competing interests?
- Do you have any non-financial competing interests in relation to this paper?

If you can answer no to all of the above, write 'I declare that I have no competing interests' below. If your reply is yes to any, please give details below.

I declare that I have no competing interests

I agree to the open peer review policy of the journal. I understand that my name will be included on my report to the authors and, if the manuscript is accepted for publication, my named report including any attachments I upload will be posted on the website along with the authors' responses. I agree for my report to be made available under an Open Access Creative Commons CC-BY license (http://creativecommons.org/licenses/by/4.0/). I understand that any comments which I do not wish to be included in my named report can be included as confidential comments to the editors, which will not be published.

Choose an item.

To further support our reviewers, we have joined with Publons, where you can gain additional credit to further highlight your hard work (see: https://publons.com/journal/530/gigascience). On publication of this paper, your review will be automatically added to Publons, you can then choose whether or not to claim your Publons credit. I understand this statement.

Yes Choose an item.
