# Supplementary material for: ISA API: An open platform for interoperable life science experimental metadata
Source: Gigascience. 2021 Sep 16;10(9):giab060. doi: 10.1093/gigascience/giab060 (PMC8444265; doi:10.1093/gigascience/giab060)
Supplement: giab060_Reviewer_2_Report_Original_Submission — Manuel Holtgrewe, PhD -- 12/18/2020 Reviewed [file giab060_reviewer_2_report_original_submission.pdf]

Reviewer Report

Title: ISA API: An open platform for interoperable life science experimental metadata

Version: Original Submission **Date:** 12/18/2020

Reviewer name: Manuel Holtgrewe, PhD

Reviewer Comments to Author:

Introduction
The authors describe the Python library "isatools" for accessing ISA (investigation study assay) files in ISA-tab and ISA-json format. The authors start by sdescribing their previous work around the ISA data model and file formats in detail. They then describe their implementation and the features of their API. They highlight the extensibility and efficiency of their object oriented model. They describe in detail how meta data can be curated in ontologies and that currently extensions are underway for the assisted creation of study meta data. They then refer to early adopters and a stable and growing community. They conclude with the statement that their library is "a major step forward in making the ISA framework open and interoperable".
General Remarks
Overall, we have found the ISA data model and ISA-tab data format to be very useful in our own work. However, there are some issues with the software including apparent bugs as described below. In 2018, my colleagues and me considered using ISA-API in our project for ISA-Tab parsing but the problems and the lack of automated tests made us roll our own (also see below).
Overall, the authors make a clear point, the paper is well-written. However, the software appears to be unfinished and some work is required to make it suitable for publication.
Major Issues
1. The ISA-creator and Bio-GraphIIn are cited as "helped grow the ISA community of users". The authors should offer evidence for this as
(a) by our own experience ISA-creator is very hard to use and this is also reflected by the expressed opinion on ISA-creator by anyone I have met so far who has used it and
(b) it is not possible to validate how Bio-GraphII has helped grow the community as the website linked to in the cited article is not available anymore and no source code is available, e.g., on Github. The Google groups forum has less than 10 threads per year, with 2 in 2020 so far and one in 2019. The authors should balance these counts with their "PyPi" download counts statistics.
2. The authors should cite other published APIs for ISA file formats, e.g., AltamISA.
- Kuhring, Mathias, et al. "AltamISA: a Python API for ISA-Tab files." Journal of Open Source Software 4.40 (2019): 1610.
3. The authors should show proof for "efficiency" of their object-oriented model, e.g., by comparing import efficiency with that of altamISA. I'm raising this point as some users raised questions on efficiency when loading/writing data files in the ISA-API Github Issues.
4. The authors write that development is in progress but it appears from the Github code frequency graph that development has mostly stalled since 2018.

5. The authors should explain in more detail how stable their API is and what the limitations and assumptions are. In my opinion, one important point in data import and export is looking how data looks after a "round-trip", e.g., import ISA-Tab, followed by export ISA-Tab.
I have done this on the official ISA data sets (https://github.com/ISA-tools/ISAdatasets, commit f20be4f83dc5f6f7ec419bfd634efba3177e4ae4). Here are the (to me unexpected results for official example data):
(a) On BII-I-1, whole columns disappear such as the first "Material Type" column,
(b) All other datasets fail to parse and parsing crashes with Python exceptions.
I think the authors should work on these points. It cannot be judged whether the software can be published this point. The software appears unfinished and some more work has to go into it to allow for publication.
Minor Issues
1. The authors should provide more automated tests for their software. In 2018 when we tried out the package we found some inconsistencies and problems but found it hard to fix bugs in the large body of software because of the lack of comprehensive automated tests.
Reviewer Signature
Manuel Holtgrewe

Level of Interest

Please indicate how interesting you found the manuscript: Choose an item.

Quality of Written English

Please indicate the quality of language in the manuscript: Choose an item.

Declaration of Competing Interests

Please complete a declaration of competing interests, considering the following questions:

- Have you in the past five years received reimbursements, fees, funding, or salary from an organisation that may in any way gain or lose financially from the publication of this manuscript, either now or in the future?
- Do you hold any stocks or shares in an organisation that may in any way gain or lose financially from the publication of this manuscript, either now or in the future?
- Do you hold or are you currently applying for any patents relating to the content of the manuscript?
- Have you received reimbursements, fees, funding, or salary from an organization that holds or has applied for patents relating to the content of the manuscript?
- Do you have any other financial competing interests?
- Do you have any non-financial competing interests in relation to this paper?

If you can answer no to all of the above, write 'I declare that I have no competing interests' below. If your reply is yes to any, please give details below.

I declare that I have no competing interests

I agree to the open peer review policy of the journal. I understand that my name will be included on my report to the authors and, if the manuscript is accepted for publication, my named report including any attachments I upload will be posted on the website along with the authors' responses. I agree for my report to be made available under an Open Access Creative Commons CC-BY license (http://creativecommons.org/licenses/by/4.0/). I understand that any comments which I do not wish to be included in my named report can be included as confidential comments to the editors, which will not be published.

Choose an item.

To further support our reviewers, we have joined with Publons, where you can gain additional credit to further highlight your hard work (see: https://publons.com/journal/530/gigascience). On publication of this paper, your review will be automatically added to Publons, you can then choose whether or not to claim your Publons credit. I understand this statement.

Yes Choose an item.
