# Supplementary material for: ISA API: An open platform for interoperable life science experimental metadata
Source: Gigascience. 2021 Sep 16;10(9):giab060. doi: 10.1093/gigascience/giab060 (PMC8444265; doi:10.1093/gigascience/giab060)
Supplement: giab060_Reviewer_3_Report_Revision_1 — Christopher Hunter, Ph.D. -- 5/12/2021 Reviewed [file giab060_reviewer_3_report_revision_1.pdf]

Reviewer Report

Title: ISA API: An open platform for interoperable life science experimental metadata

Version: Revision 1 **Date:** 5/12/2021

Reviewer name: Christopher Hunter, Ph.D.

Reviewer Comments to Author:

Review of GIGA-D-20-00333 R1
Full Title: ISA API: An open platform for interoperable life science experimental metadata
The manuscript is well written and coherent, it provides a nice balance of historical context of ISA-Tools and the current release of the ISA-API.
As a biologist and Biocurator I can attest to the importance of simple to use tools for curation of datasets, and the ISA-creator has been well used by the community over the years. The addition of the ISA-API should allow for more repositories to incorporate the use of ISA formats as both import and export formats for datasets. I have to admit that my lack of experience as a developer means that I am in no position to actually test the API's functionality so I cannot comment as to the technical suitability of the implementation or even whether is works or not!
I have been requested to review the manuscript with specific reference to the original reviewer 2 concerns:
Reviewer 2 Comment 1"The ISA-creator and Bio-GraphIIn are cited as "helped grow the ISA community of users". The authors should offer evidence for this as;
(a) by our own experience ISA-creator is very hard to use and this is also reflected by the expressed opinion on ISA-creator by anyone I have met so far who has used it and
(b) it is not possible to validate how Bio-GraphII has helped grow the community as the website linked to in the cited article is not available anymore and no source code is available, e.g., on GitHub. The Google groups forum has less than 10 threads per year, with 2 in 2020 so far and one in 2019. The authors should balance these counts with their "PyPi" download counts statistics."
My comment: I believe the authors have addressed the primary concern about the evidence of continued growth in the ISA user community with the detailed description of the PyPi download statistics. The issue of ISA-creators user experience by the reviewer and anecdotal comment of all who have used it, is unfounded and in-fact if true, adds to the argument for the implementation of the ISA-API as a means to allow a wider developer-base to improve the ISA-creation experience.
Reviewer 2 comment 2. "The authors should cite other published APIs for ISA file formats, e.g., AltamISA."
- Kuhring, Mathias, et al. "AltamISA: a Python API for ISA-Tab files." Journal of Open Source Software 4.40 (2019): 1610.
My comment: The authors have made appropriate changes and included the suggested reference.
Reviewer 2 comment 3. "The authors should show proof for "efficiency" of their object-oriented model, e.g., by comparing import efficiency with that of AltamISA. I'm raising this point as some users raised questions on efficiency when loading/writing data files in the ISA-API GitHub Issues."

My comment: The authors have replaced the word efficiency with coherent in the manuscript to clarify the meaning in the relevant paragraph. However I'm not sure they have addressed the principle of the concern raised by reviewer 2, i.e. how does the ISA-API compare to other existing models in terms of efficiency? As I have no idea how to measure "efficiency" of a model I'm not convinced this is a valid request from reviewer 2.
Reviewer 2 comment 4. "The authors write that development is in progress but it appears from the GitHub code frequency graph that development has mostly stalled since 2018."
My comment: I agree with the authors rebuttal of this point, simply looking at GitHub commits is not a suitable measure.
Reviewer 2 comment 5. "The authors should explain in more detail how stable their API is and what the limitations and assumptions are. In my opinion, one important point in data import and export is looking how data looks after a "round-trip", e.g., import ISA-Tab, followed by export ISA-Tab.
I have done this on the official ISA data sets (https://github.com/ISA-tools/ISAdatasets, commit f20be4f83dc5f6f7ec419bfd634efba3177e4ae4). Here are the (to me unexpected results for official example data):
(a) On BII-I-1, whole columns disappear such as the first "Material Type" column,
(b) All other datasets fail to parse and parsing crashes with Python exceptions."
My comment: Unfortunately my lack of the required skill set to make any sort of tests myself means I am not in a position to adjudicate on this point! I do agree with the authors rebuttal that they cannot assess the reviewers issues based on the minimal information provided in the review. As the authors point out, documentation can always be improved, and 1 such improvement might be to include a "round-trip" example as the reviewer 2 has attempted to show that one can take a valid ISA formatted input, convert it to say SRA format, and back to ISA format using the API and that the input and output ISA formats do indeed match.
Reviewer 2 comment Minor issue 1. "The authors should provide more automated tests for their software. In 2018 when we tried out the package we found some inconsistencies and problems but found it hard to fix bugs in the large body of software because of the lack of comprehensive automated tests."
My comment: I think this reviewers comment is un-related to the review, they are talking about a version of the tool that is approximately 3 years old, not the current version that they are meant to be reviewing. Despite the irrelevance, the authors have responded by adding text to highlight the Test Driven Development approach taken in the project.
With the one caveat already mentioned, i.e. I am unable to actually test the code so I am reliant on the other reviewer to have covered that aspect of the review, I believe the manuscript is suitable for publication as the authors have adequately addressed all of the reviewer 2 comments with the possible exception of improved documentation.

Level of Interest

Please indicate how interesting you found the manuscript: Choose an item.

Quality of Written English

Please indicate the quality of language in the manuscript: Choose an item.

Declaration of Competing Interests

Please complete a declaration of competing interests, considering the following questions:

- Have you in the past five years received reimbursements, fees, funding, or salary from an organisation that may in any way gain or lose financially from the publication of this manuscript, either now or in the future?
- Do you hold any stocks or shares in an organisation that may in any way gain or lose financially from the publication of this manuscript, either now or in the future?
- Do you hold or are you currently applying for any patents relating to the content of the manuscript?
- Have you received reimbursements, fees, funding, or salary from an organization that holds or has applied for patents relating to the content of the manuscript?
- Do you have any other financial competing interests?
- Do you have any non-financial competing interests in relation to this paper?

If you can answer no to all of the above, write 'I declare that I have no competing interests' below. If your reply is yes to any, please give details below.

I am a current employee of GigaScience Press, but I have performed the review with no bias and I have no personal gain/loss that will result from publication (or not) of this manuscript.

I agree to the open peer review policy of the journal. I understand that my name will be included on my report to the authors and, if the manuscript is accepted for publication, my named report including any attachments I upload will be posted on the website along with the authors' responses. I agree for my report to be made available under an Open Access Creative Commons CC-BY license (http://creativecommons.org/licenses/by/4.0/). I understand that any comments which I do not wish to be included in my named report can be included as confidential comments to the editors, which will not be published.

Choose an item.

To further support our reviewers, we have joined with Publons, where you can gain additional credit to further highlight your hard work (see: https://publons.com/journal/530/gigascience). On publication of this paper, your review will be automatically added to Publons, you can then choose whether or not to claim your Publons credit. I understand this statement.

Yes Choose an item.
